# Sustainable Extraction of *Hedera helix* Bioactive Compounds via Synergy of Natural Deep Eutectic Solvent and Ultrasound: Process Optimization, Mechanistic Insights and Anti-Colon Cancer Activity

**DOI:** 10.3390/ijms27052295

**Published:** 2026-02-28

**Authors:** Tangtang Li, Kai Hua, Shuya Ye, Juzhao Liu, Hongliang Chen, Yiming Sun, Xiaoxia Shen, Qi Cui

**Affiliations:** School of Pharmaceutical Sciences, Zhejiang Chinese Medical University, Hangzhou 311402, China

**Keywords:** *Hedera helix*, natural deep eutectic solvents, ultrasound-assisted extraction, anti-colon cancer, reactive oxygen species

## Abstract

*Hedera helix* (HE) contains diverse bioactive constituents, including triterpenoid saponins, flavonoids, and phenolic acids, which exhibit various pharmacological activities. In this study, ultrasound-assisted extraction (UAE) combined with natural deep eutectic solvent (NADES) was employed to enhance the extraction efficiency and elucidate the underlying mechanisms. Among the tested formulations, a ternary system composed of malonic acid (Mal), N,N′-dimethylurea (DMU), and 1,4-butanediol (1,4-BDO) achieved the highest efficiency for extracting eight target compounds from the HE leaves. In addition, the key interactions among NADES components were confirmed by Fourier-transform infrared (FT-IR) spectroscopy, providing valuable insights into the extraction mechanism. The UAE process was systematically optimized through single-factor experiments. Subsequently, response surface methodology (RSM) identified the optimal conditions as ultrasonic time of 45 min, solid/liquid ratio of 1:54 g/mL, and ultrasonic temperature of 42 °C. Scanning electron microscopy (SEM) elucidated the microstructural alterations in plant cell walls induced by NADES-UAE, alongside the enhanced penetration and disruption mechanisms. In vitro bioactivity revealed that the NADES-extracted HE exerted strong inhibitory effect on HT-29 colon cancer cells. Overall, these findings demonstrate the high effectiveness and sustainability of NADES-UAE for extracting HE bioactive compounds and provide valuable implications for the industrial-scale production of plant-based functional products.

## 1. Introduction

*Hedera helix* (HE), an evergreen perennial climber in the genus Hedera (Araliaceae), is native to continental Europe and western Asia [1]. Beyond its ornamental value, HE is cultivated as a source of high-value bioactive compounds for diverse applications. Its leaves (HEL) possess significant medicinal properties and are recognized in both the European Pharmacopoeia and British Pharmacopoeia for treating cough, asthma, and bronchial ailments [2]. HEL contains several classes of bioactive compounds, including triterpenoid saponins (hederacoside C (HDC), α-hederin (α-H), hederacoside B (HDB), and hederacoside D (HDD)), flavonoids (rutin (RU) and nicotiflorin (NCF)), and phenolic acids (chlorogenic acid (CHA) and cryptochlorogenic acid (CRA)). These compounds exhibit diverse pharmacological activities: saponins demonstrate antitumor, antiproliferative, and antispasmodic effects; flavonoids suppress angiogenesis and regulate rheumatic diseases biomarkers; and phenolic acids show antibacterial, antioxidant, and anti-inflammatory properties [3,4,5,6,7]. Consequently, efficient extraction of HEL bioactive compounds is crucial for pharmaceutical development.

Conventional extraction of HEL saponins, flavonoids, and phenolic acids predominantly focuses on organic solvents like methanol (MeOH) and ethanol (EtOH) [6,8]. Nevertheless, these solvents pose safety hazards and environmental risks, including greenhouse gas emissions and aquatic pollution, which contradict the principles of sustainable development [9]. Consequently, reducing the use of hazardous solvent use and developing environmentally benign alternatives is imperative [10,11]. In this context, “natural deep eutectic solvent (NADES)”, introduced in 2011, presents a promising biodegradable and efficient alternative [12]. As a novel derivative of deep eutectic solvents (DESs), NADES comprise binary or multicomponent systems of natural metabolites (e.g., glucose, sucrose, proline, mannitol) sourced from living organisms, offering exceptional biocompatibility and sustainability [13,14,15]. Their formation involves hydrogen bond acceptors (HBAs) and donors (HBDs), with strong charge-transfer interactions that significantly lower the system’s melting point [16]. NADES exhibit multiple advantages, including excellent biodegradability, low cost, tunable polarity, antimicrobial activity, and simple synthesis [17]. Currently, NADES-mediated extraction demonstrates broad applicability for isolating bioactive compounds from natural matrices, with recent studies confirming their efficacy and versatility [18,19,20,21]. Recent studies have significantly advanced the field of NADES through mechanistic innovations and application expansions. It is confirmed that hydrogen bonding is the key mechanism in NADES-flavonoid interactions using multispectral analysis [22]. Wu et al. [23] developed multi-target biopesticides by efficiently extracting gossypol derivatives from cotton byproducts. Wang et al. [24] achieved high-stability extraction of labile phenolics, while Singh et al. [25] enabled targeted metabolite enrichment from diverse geographical plants for improved herbal quality control. These latest developments highlight the evolving sophistication of NADES design for precision extraction. Despite this potential, the systematic exploration of NADES for the efficient extraction of HEL bioactive compounds remains unexplored. Given their exceptional green credentials, developing targeted NADES extraction protocols for HEL could significantly advance sustainable phytochemistry. Of particular significance, ternary deep eutectic solvents (TDESs) exhibit markedly enhanced extraction efficiency and selectivity relative to their binary counterparts [26]. To bridge this technological gap, we have developed a malonic acid (Mal)-N,N′-dimethylurea (DMU)-1,4-butanediol (1,4-BDO) TDES tailored for HE extraction. Within this formulation, Mal, as a potent hydrogen-bond donor, DMU establishes complementary hydrogen-bond acceptance sites, and 1,4-BDO serves to reduce viscosity while promoting mass transfer. This harmonized solvent system achieves efficient concurrent extraction of medium-polarity triterpenoid saponins alongside more polar phenolic constituents.

Plant bioactive compounds are commonly extracted using methods including heating reflux extraction (HRE), cold soaking extraction (CSE), microwave-assisted extraction (MAE), supercritical fluid extraction (SFE), and ultrasound-assisted extraction (UAE) [27,28,29,30,31,32]. Among these, the UAE is widely employed, utilizing high-frequency mechanical waves to induce cavitation. This phenomenon facilitates interparticle collisions and cellular disruption, significantly enhancing the dissolution efficiency and bioavailability of target compounds [33]. Therefore, in comparison to conventional extraction techniques, UAE significantly reduces processing duration and energy consumption while achieving superior yields of target compounds. Its mild operating conditions, particularly the low-temperature environment, are highly conducive to preserving the structural integrity and bioactivity of thermolabile constituents, such as flavonoids and phenolic compounds [34,35,36]. Recognized as a pivotal green extraction strategy, the UAE demonstrates further enhanced efficiency when coupled with NADES [18,37]. The NADES-UAE system enables selective enrichment of bioactive compounds, improving recovery yields while reducing processing time [38,39]. NADES-UAE technology has been successfully applied for the efficient extraction of various bioactive compounds from plants. Typical applications include: the optimized extraction of trans-anethole from fennel seeds using DES with improved yield through kinetic modeling, and the highly efficient extraction of crocin from saffron by combining cryogenic grinding with NADES, significantly enhancing extraction efficiency through process optimization [40,41]. This technology is also widely used for the extraction of flavonoids from sweet tea, polyphenols from *Mentha pulegium*, and the simultaneous extraction of crocin and geniposide from *Gardenia jasminoides* fruits [42,43,44,45]. Although significant progress has been made in the extraction of certain plant materials, current methods for HE still predominantly rely on conventional solvents (e.g., MeOH and EtOH) and traditional techniques such as reflux heating extraction. Currently, no environmentally sustainable and efficient extraction methodologies, such as NADES-UAE, have been implemented in the industrial production of HE [46]. Furthermore, existing research on *Hedera helix* has primarily focused on individual compound classes (e.g., saponins or flavonoids), overlooking the synergistic potential of multi-constituent extraction. More critically, the mechanistic understanding of solvent-plant matrix interactions during extraction remains poorly elucidated, particularly for sustainable solvent systems.

To address these challenges, this study introduced a novel NADES-UAE integrated approach for the simultaneous extraction of eight bioactive compounds (CHA, CRA, RU, NCF, HDC, HDB, HDD, α-H) from HEL. The strategic selection of NADES formulations was designed not only to align with green chemistry principles but also to achieve high extraction efficiency. The methodology comprised: (1) screening of NADES types based on target compound content, (2) optimization of molar ratio and water content, and (3) systematic enhancement of UAE parameters through single-factor experiments and Box–Behnken Design (BBD) coupled with Response Surface Methodology (RSM). The NADES-UAE extracts were evaluated for their anti-HT-29 colon cancer activity using Cell Counting Kit-8 (CCK-8) assay and flow cytometry, as well as for their antioxidant capacity. Therefore, the present study aimed to establish a sustainable extraction platform for HEL, to evaluate its potential as a source of high-value bioactive compounds for potential industrial applications in the pharmaceutical and cosmetic sectors.

## 2. Results and Discussion

### 2.1. Results of Active Ingredient Content Determination

**Figure 1:**
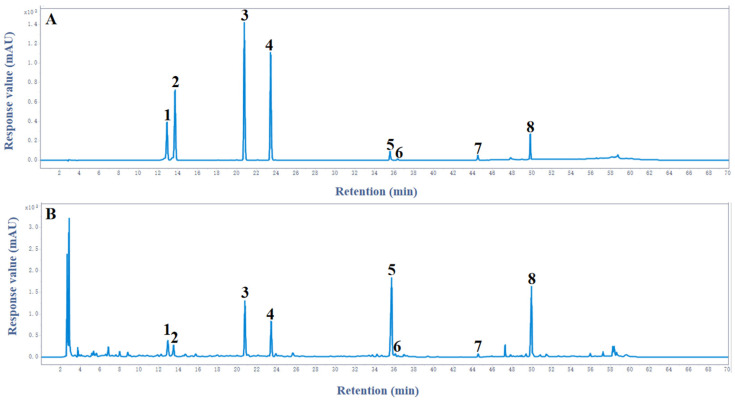
HPLC chromatograms of reference standards (**A**) and *Hedera helix* leaves extract (**B**): (1) chlorogenic acid, (2) cryptochlorogenic acid, (3) rutin, (4) nicotiflorin, (5) hederacoside C, (6) hederacoside D, (7) hederagenin B, and (8) α-hederin.

A robust HPLC method was developed and validated in this study for the simultaneous determination of eight bioactive compounds in HEL extracts, namely CHA, CRA, RU, NCF, HDC, HDD, HDB, and α-H. As illustrated in Figure 1, the optimized chromatographic conditions enabled excellent baseline separation of all target analytes within a single analytical run. As shown in Table 1, the method exhibited superior analytical performance. Specifically, (1) high sensitivity was achieved, with limits of detection ranging from 0.014 to 2.900 μg/mL; (2) calibration curves demonstrated excellent linearity with strong correlation coefficients (R^2^ > 0.999) across all analytes (CHA (y = 15,917.08x − 114.60, R^2^ = 0.9998), CRA (y = 14,971.00x − 32.60, R^2^ = 0.9992), RU (y = 29,350.11x + 1558.52, R^2^ = 0.9994), NCF (y = 27,395.79x + 344.80, R^2^ = 0.9993), HDC (y = 2104.43x + 101.78, R^2^ = 0.9999), HDD (y = 2158.50x + 53.70, R^2^ = 0.9991), HDB (y = 2172.90x + 46.58, R^2^ = 0.9991), α-H (y = 3360.99x + 29.70, R^2^ = 0.9999), where y represents peak area and x represents concentration, μg/mL); (3) precision was excellent (RSD ≤ 1.201%) with high repeatability (RSD ≤ 1.985%); and (4) recovery rates ranged from 97.949% to 102.783%, confirming outstanding accuracy. Therefore, this HPLC method provides a reliable analytical platform for holistic quality assessment of HE-derived nutraceuticals. Compared with existing methods, this protocol exhibits significant advantages, including a simplified sample preparation process and improved chromatographic resolution of structurally similar saponins. These improvements are highly relevant for establishing robust quality control measures in the industrial production of plant-based products.

### 2.2. Optimization of NADES

#### 2.2.1. Selection of Optimal NADES Formulation

**Figure 2:**
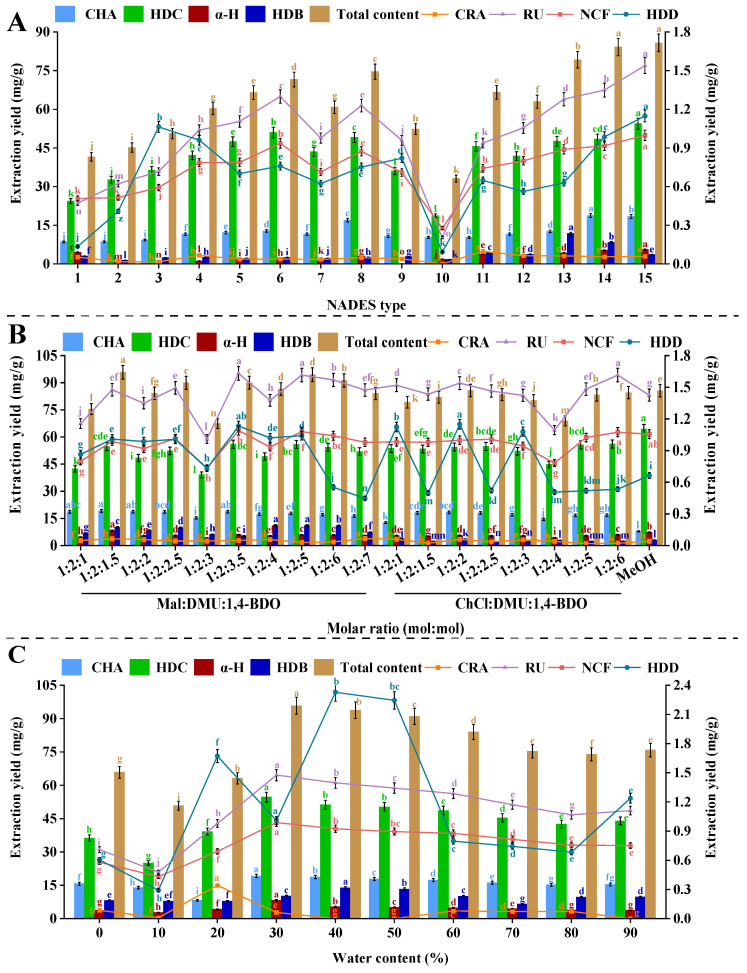
Effects of different NADES formulations (**A**), molar ratios (**B**), and water contents (**C**) on extraction yields of eight bioactive compounds from *Hedera helix*. Data are presented as mean ± SD (*n* = 3). Different lowercase letters (a–o) indicate statistically significant differences (*p* < 0.05, Tukey’s test).

Based on the synergistic interactions between HBDs and HBAs, NADES exhibited unique physicochemical properties and formed three-dimensional intermolecular hydrogen-bonding networks, which could govern their polarity, viscosity, and solvation capacity [47,48]. ChCl and Bet, as cost-effective and renewable resources, exhibit higher extraction efficiency compared to other types of NADES [49]. Additionally, preliminary experiments revealed that Mal serves as an effective HBA. Therefore, ChCl, Bet, and Mal were selected as HBAs, while urea, glucose, organic acids, and DMU were employed as HBDs. Notably, the ternary ChCl-Ur-Mal system demonstrated exceptional polyphenol extraction efficiency (Figure 2A), consistent with previous reports on the recovery of polyphenol using polar NADES [50]. Extraction efficiency strongly correlated with functional group polarity (carboxyl > amine > glycosyl > hydroxyl), favoring highly polar phenolic acids (CHA and CRA) structurally analogous to Mal [51]. Based on protonation-mediated solute-solvent interactions, acidic NADES formulations could enhance the phenolic compounds solubility. As the most polar HBA, ChCl could strengthen the hydrogen bonding with phenolic hydroxyl groups, thus significantly enhancing the yield. Based on these findings, the application of Mal-DMU-1,4-BDO (1:2:2) and ChCl-DMU-1,4-BDO (1:2:2) as optimal systems for phenolic extraction was further validated, which could underscore the crucial functions of HBD-HBA complementarity in designing task-specific solvents.

Compared with ChCl-1,4-BDO (1:2) and Mal-DMU-1,4-BDO (1:2:2), the ChCl-DMU-1,4-BDO (1:2:2) system demonstrated superior flavonoid extraction efficiency for RU and NCF, and this performance hierarchy could be attributed to three synergistic mechanisms. Under the ChCl-mediated alkaline conditions, RU and NCF acquired a pronounced negative charge, which could enhance the hydrophilicity and NADES compatibility through charge-mediated interactions [52]. As evidenced by recent studies, multiple hydroxyl groups in flavonoids could form directional hydrogen bonds with ChCl-DMU-1,4-BDO (1:2:2) components, which could indicate the superior flavonoid extraction performance of alcohol-based NADES systems based on their high hydrogen-bonding capacity and optimal polarity alignment with target analytes [53]. In addition, the ChCl-mediated alkaline environment could enhance flavonoid deprotonation, while complementary hydrogen-bonding sites were provided by polyols, which synergistically enhanced the binding affinity versus the acidic NADES formulations. These findings are in agreement with the established structure-NADES relationships for phenolic compound extraction, which further confirms the crucial functions of charge state and polarity matching in designing green solvents.

ChCl-DMU-1,4-BDO (1:2:2) exhibited optimal extraction efficiency for saponin-like constituents (HDC, HDD, HDB, and α-H), which significantly outperformed Mal-Ur-1,4-BDO (1:2:2) and Mal-DMU-1,4-BDO (1:2:2). These findings were consistent with previous reports, which highlighted the improved saponin solubility under weakly acidic conditions [54]. Specifically, the superior performance could be attributed to the weakly acidic properties of the ChCl-Mal-based NADES system. This solvent combination exhibited low surface tension and superior matrix penetration capability, both of which could contribute to the effective solubilization of saponins. Additionally, the optimal polarity and hydrogen-bonding capacity of ChCl-DMU-1,4-BDO (1:2:2) could further promote interactions with the glycosylated structures of saponins, thus maximizing the extraction yield. In addition, significant differences in total extraction yields could be observed in a comprehensive evaluation of various NADES formulations. Notably, Mal-DMU-1,4-BDO (1:2:2) and ChCl-DMU-1,4-BDO (1:2:2) demonstrated superior performance, achieving 2.895–2.897 folds higher total compound yields versus the least effective Bet-DMU (1:2). This marked difference in efficiency could be attributed to the distinct hydrophilic properties of these solvents, in which Mal-DMU-1,4-BDO (1:2:2) and ChCl-DMU-1,4-BDO (1:2:2) exhibited exceptional extraction capacity based on their pronounced hydrophilicity, which could promote robust solute-solvent interactions through multiple hydrogen bonding sites. Therefore, the above phenomena could facilitate the enhanced solvation and mass transfer of bioactive compounds. In contrast, weaker hydrogen bonds were formed by Bet-DMU (1:2), which resulted in suboptimal solvent penetration and reduced extraction efficiency. Based on the above findings, Mal-DMU-1,4-BDO (1:2:2) and ChCl-DMU-1,4-BDO (1:2:2) were selected for further optimization studies to maximize the bioactive compound extraction performance.

The distinct extraction preferences observed among different compound classes provide direct evidence for the critical role of structural features in NADES selectivity (Figure S1). Phenolic acids and flavonoids, characterized by abundant hydroxyl and carboxyl groups, exhibited higher affinity for ChCl- and Mal-based NADES, while saponins with varying sugar chain lengths showed differential partitioning behavior. These structure-dependent interactions originate from the hydrogen-bonding patterns between specific functional groups of the target compounds and the solvation components of NADES. To theoretically elucidate the cell wall-disrupting capability of the NADES systems and quantitatively understand this structure–selectivity relationship, the Kamlet–Taft solvation parameters, namely hydrogen bond basicity (β), hydrogen bond acidity (α), and dipolarity/polarizability (π*), were introduced. The selection of these parameters was grounded in their well-defined physicochemical significances, which are directly relevant to the key interactions during extraction. A high β value, representing strong hydrogen bond acceptance, is crucial for breaking the extensive hydrogen bonding network within the cell wall (e.g., between cellulose and hemicellulose), thereby inducing structural degradation. The α value, indicating hydrogen bond donor capacity, influences specific interactions with polar functional groups of cell wall components. Meanwhile, a low π* value reflects a less polar environment, which enhances the solvent’s affinity for hydrophobic entities, such as the aglycone moieties of saponins, thereby promoting their solvation and release. As shown in Figure S2, these parameters for different NADES systems were measured by 4-Nitroaniline, N,N-diethyl-4-nitroaniline, and Reichardt’s dye. Consistent with our rationale, the α values of the acid-based NADES could not be measured, while the other types of NADES ranged from 0.54 to 1.02. The basic-based NADESs exhibited relatively high β values, confirming their potent capability to break down the hydrogen-bonding matrix of the cell wall. Furthermore, Mal-DMU-1,4-BDO (1:2:2) and ChCl-DMU-1,4-BDO (1:2:2) displayed distinctly small π* values, underscoring their superior ability to non-specifically interact with hydrophobic components. This unique combination of properties-high hydrogen bond basicity for cell wall disruption and low polarity for hydrophobic domain penetration-provides a compelling explanation for the high extraction efficiency. Consequently, Mal-DMU-1,4-BDO (1:2:2) and ChCl-DMU-1,4-BDO (1:2:2) effectively promote the release of bioactive components through a synergistic mechanism of cell wall degradation and enhanced solvation of target components.

#### 2.2.2. Molar Ratio of NADES

Based on the modulation of the hydrogen bond network density and solvent viscosity through the adjustment of the HBA/HBD molar ratio, the extraction yield was systematically optimized. Building upon preliminary screening results (identified Mal-DMU-1,4-BDO and ChCl-DMU-1,4-BDO as optimal solvents), an extensive investigation with the application of ten distinct molar ratio formulations (ranging from 1:2:1 to 1:2:7 in 0.5 increments) was conducted in this study, in which the experimental design allowed for meticulous characterization of the structure-property relationship between solvent composition and extraction performance. Compared with ChCl-DMU-1,4-BDO, it was found in the systematic evaluation that various NADES14 formulations exhibited consistently superior extraction efficiency for all eight target components (Figure 2B), which might be attributed to the stronger hydrogen bonding capacity of Mal-DMU-1,4-BDO, where the carboxyl groups of Mal could form particularly robust interactions with the active components. Functionally, the electron-rich nature of these carboxyl moieties could facilitate more effective hydrogen bonding with polar functional groups (hydroxyl, carbonyl, etc.) in the target compounds, which contributed to the significant improvement of their solubilization and extraction yield. It was demonstrated that 1,4-BDO content in NADES14 exhibited a clear positive correlation with the extraction yields of phenolic compounds, flavonoids, and saponins. Notably, the maximal total extraction yield of all eight constituents was achieved at the optimal 1:2:1.5 molar ratio. These results were in agreement with the findings of Sa et al. [55], demonstrating that the increase of 1,4-BDO content could enhance hydroxyl group density, which in turn improved the hydrogen-bond-mediated solubilization of target constituents. However, Mal-DMU-1,4-BDO exhibited significantly reduced extraction efficiency when the 1,4-butanediol ratio exceeded 1.5, which might be mechanistically attributed to the excessive viscosity caused by the extended hydrocarbon chain of 1,4-BDO, which could impede the mass transfer and ultimately compromise the component dissolution.

Consistent with previously reported physicochemical characteristics of NADES systems [56]. As presented in Table 2, all NADES formulations demonstrated significantly higher viscosities (16.640–21.600 mPa·s) than conventional solvents, including water (1.002 mPa·s) and methanol (0.560 mPa·s), providing a physicochemical basis for their enhanced extraction capabilities observed in Figure 2B. A positive correlation between viscosity and extraction performance was observed within an optimal range. Increasing the Mal-DMU-1,4-BDO ratio from 1:2:1 to 1:2:1.5 resulted in a viscosity elevation from 16.640 to 18.646 mPa·s, which corresponded to improved extraction yields for all eight target compounds. This enhancement can be attributed to the strengthened hydrogen-bonding network among the NADES components, which promotes mass transfer and facilitates the release of bioactive constituents from the plant matrix [57]. However, exceeding the optimal viscosity threshold adversely affected extraction efficiency [40]. Further increase in the 1,4-BDO proportion to a 1:2:2 ratio elevated the viscosity to 21.600 mPa·s, likely due to the extended hydrocarbon chains creating greater molecular entanglement. This excessive viscosity impeded solvent diffusion and increased mass transfer resistance, ultimately reducing extraction yields. Therefore, the Mal-DMU-1,4-BDO system at a 1:2:1.5 molar ratio represents the optimal balance between hydrogen-bonding capacity and viscosity-controlled mass transfer efficiency.

**Figure 3:**
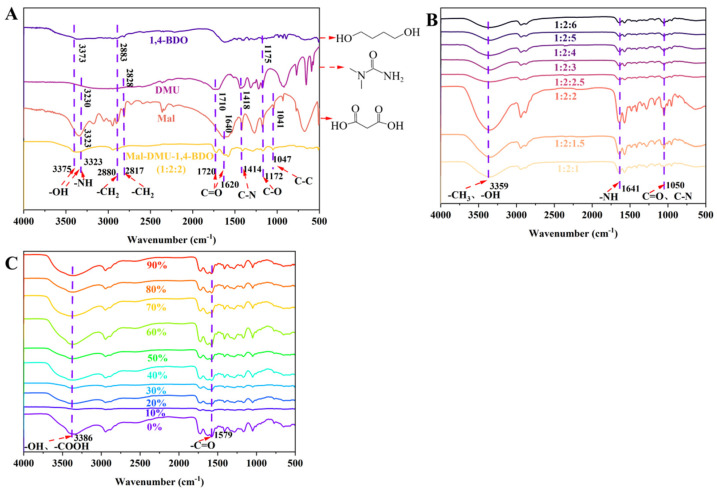
Comparative analysis of FT-IR spectral features of the eutectic system NADES14 and its molecular constituents (**A**), Mal:DMU:1,4-BDO at different molar ratios (**B**) and Mal:DMU:1,4-BDO = 1:2:1.5 (mol:mol) with different water content (**C**).

**Figure 4:**
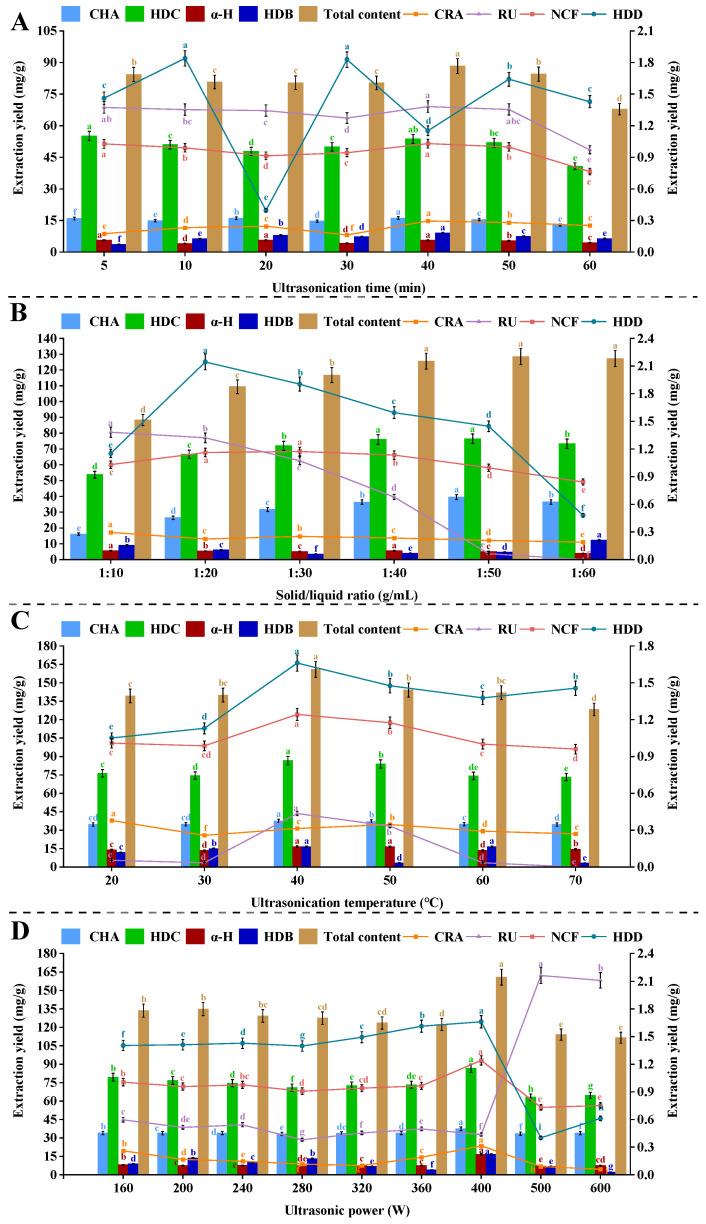
Effects of ultrasonic time (**A**), ultrasonic temperature (**B**), ultrasonic power (**C**), and solid/liquid ratio (**D**) on extraction yields of eight bioactive components from *Hedera helix*. Data are presented as mean ± SD (*n* = 3). Different lowercase letters (a–h) indicate statistically significant differences (*p* < 0.05, Tukey’s test).

NADES represent a novel class of designer solvents formed through hydrogen-bond-mediated self-assembly of two or more constituents, which exhibit significantly depressed melting points compared to their components [58]. In this work, Fourier-transform infrared spectroscopy (FT-IR, Nicolet IS50, Thermo Fisher Scientific, Waltham, WA, USA) was employed to elucidate the molecular interactions in the prepared NADESs (Figure S3 and Figure 4), in which the optimized Mal-DMU-1,4-BDO formulation (1:2:1.5, molar ratio) was applied as an example [59]. Specifically, FT-IR spectra were acquired at ambient temperature (25 ± 1 °C) across the mid-infrared region (4000–400 cm^−1^) with a resolution of 4 cm^−1^. As shown in Figure 3, the spectrum of Mal-DMU-1,4-BDO displayed several characteristic features. For instance, a broad O-H stretching band was observed at 3375 cm^−1^, which corresponded to the intensive hydrogen bonding between the carboxyl group (-COOH) of Mal and the hydroxyl group (-OH) of 1,4-BDO. In addition, a significant red-shift of carbonyl stretching vibrations was observed from 1640 cm^−1^ (Mal) and 1710 cm^−1^ (DMU) to 1620 cm^−1^ in the NADES system. The pronounced broadening of the O-H stretching band could serve as the definitive evidence for NADES formation, which further confirms the successful establishment of extensive three-dimensional hydrogen-bonding networks. Notably, these interactions could enhance solvent polarity, and they could account for the marked depression of the melting point relative to individual components. Additionally, the observed red-shift in carbonyl stretching vibrations demonstrated the reduced bond strength due to the formation of a hydrogen bond between NADES constituents. Overall, these spectroscopic findings could provide molecular-level evidence for the systematic optimization results, in which Mal-DMU-1,4-BDO at a 1:2:1.5 molar ratio was identified as the optimal formulation for maximizing extraction efficiency.

#### 2.2.3. Water Content in NADES

According to previous studies, NADES exhibited a unique synergistic effect through hydrogen bond formation with water molecules, which could facilitate the solubilization of target bioactive compounds [60]. Notably, the incorporation of water exhibited three crucial functions during the extraction process: (1) induction of cellular expansion and wall disruption to improve NADES permeability, (2) reduction in solvent viscosity to optimize mass transfer kinetics, and (3) modulation of solvent polarity to enhance the phytochemical solubility. Based on the systematic investigation of water content (0–90%, *v*/*v*), distinct hydration-dependent extraction phases were identified in this study (Figure 2C), in which the limited extraction efficiency under low water content (0–20%) could be primarily attributed to two factors: incomplete formation of hydrogen bond networks between NADES components and water molecules, alongside the enhanced solvent viscosity that restricted the molecular mobility. As evidenced by relatively lower extraction yields versus optimal conditions, these conditions collectively resulted in suboptimal solute-solvent interactions [61]. The apex of extraction performance occurred at 30% water content, where Mal-DMU-1,4-BDO (1:2:1.5) achieved maximum extraction efficiency, and it could be attributed to the reduced solvent viscosity that enhanced mass transfer kinetics, alongside the enhanced polarity that improved the solvation of target compounds [62]. Additionally, this optimal hydration level could contribute to an ideal balance between the intactness of the hydrogen-bond network and sufficient fluidity, which is aimed at efficient extraction. However, progressive yield reduction for flavonoids, phenolics, and saponins would occur above this critical threshold, which could be attributed to the excessive water molecules that competed with target compounds for hydrogen bonding sites in the NADES network, and disrupted the molecular architecture of the solvent [63]. In addition, it was revealed by the comparative studies that this phenomenon was system-dependent, and the optimal water content exhibited a varying trend with the changes in NADES formulation and target compounds. For instance, dogwood fruit antioxidants obtained the maximal recovery at 50% (*v*/*v*) water content. It could be demonstrated in our systematic optimization that 30% water content represented the precise equilibrium point for Mal-DMU-1,4-BDO (1:2:1.5), in which the hydrogen bonding capacity and solvent fluidity reached an optimal balance for phytochemical extraction. Overall, these findings could provide crucial guidance for further development of efficient NADES-based extraction protocols, emphasizing the necessity for formulation-specific water content optimization.

To further elucidate the mechanistic role of water content in the Mal-DMU-1,4-BDO (1:2:2) system, FT-IR analysis was conducted across a hydration gradient (0–90%) under consistent extraction conditions. As illustrated in Figure 3C, increasing the water content from 0% to 30% resulted in a pronounced redshift and broadening of the O–H stretching band centered at 3386 cm^−1^, indicating that water molecules actively participated in the reorganization of the hydrogen-bonding network within the NADES system through the formation of intermolecular hydrogen bonds. Concurrently, systematic variations were observed across other functional group regions. The C=O stretching vibration (~1700 cm^−1^) shifted from 1705 cm^−1^ to 1579 cm^−1^; this substantial shift suggests the establishment of strong hydrogen-bonding interactions between water molecules and the carbonyl groups of NADES components. The diminished intensity of the C–H stretching vibration (~2900 cm^−1^) implies a reorganization of the microenvironment surrounding the alkyl chains. Furthermore, the amide I (~1640 cm^−1^) and amide II (~1550 cm^−1^) bands of the DMU component exhibited progressive broadening and attenuation, confirming competitive hydrogen bonding with water molecules that disrupted the original hydrogen-bond network within the NADES. In the fingerprint region (1000–1500 cm^−1^), although no distinct new bands emerged, a gradual decrease in absorption intensity with increasing water content was observed, suggesting an indirect influence of water molecules on the skeletal vibrations and conformational dynamics of the NADES components. This hydration-induced spectral shift phenomenon is consistent with observations reported by Tian, Wu, and Sakurai et al. in analogous NADES systems, who documented that hydration induces a shift in the O–H stretching band toward higher wavelengths [64,65,66]. This spectral behavior provides direct evidence of water-mediated reorganization and reinforcement of the hydrogen-bonding network, as established in the foundational work of Gabriele et al. [67]. The most significant spectral modifications occurred at 30% hydration, correlating precisely with the peak extraction efficiency shown in Figure 2C. This synergy indicates that the optimized hydrogen-bonding capacity at this hydration level enhances cell wall disruption and compound solubilization in HE.

Conversely, when water content exceeded 30% (40–90%), the FT-IR profiles progressively converged toward that of pure water, demonstrating progressive dilution of the NADES-specific molecular architecture and a corresponding attenuation of hydrogen-bonding interactions, as quantified in studies by Filip et al. [68]. This structural transition correlates directly with the declining extraction efficiency for all eight target compounds (Figure 2C), confirming that excessive hydration diminishes the solvent’s unique solubilizing properties. The FT-IR evidence thus provides a coherent mechanistic rationale for the observed optimal extraction performance at 30% water content, highlighting the critical balance between hydrogen-bonding enhancement and structural integrity in NADES-mediated extraction.

### 2.3. Single-Factor Experiment Results

#### 2.3.1. Ultrasonic Time

Ultrasonic time serves as a critical parameter in UAE optimization, where an optimal duration could maximize the NADES extraction yield with minimal energy consumption [69,70]. In this research, the extraction kinetics of eight target components were systematically evaluated across a gradient of ultrasonic time (5–60 min), in order to determine the optimal extraction time. For further experiments, the extraction was carried out using the most effective solvent selected previously (Mal-DMU-1,4-BDO), under the following extraction parameters: ultrasonic time (40 min), ultrasonic temperature (50 °C), solid/liquid ratio (1:50 g/mL), and ultrasonic power (400 W). As illustrated in Figure 4A, the initial extraction phase (5–30 min) exhibited relatively low yields, which might be attributed to the insufficient cavitation energy for effective cell wall disruption, alongside the limited solvent penetration through intact cellular membranes [70]. In addition, this phenomenon was consistent with the proposed mechanisms, in which the pronounced concentration gradient between Mal-DMU-1,4-BDO (1:2:1.5) and intracellular bioactive components could drive the solvent diffusion and osmotic effects, thus facilitating the dissolution of target constituents. Furthermore, the cavitation effects were intensified by the prolonged ultrasonication, which could progressively disrupt the plant cell walls and mitigate the mass transfer limitations imposed by cellular structures. Concurrently, microstreaming and microturbulence were generated by bubble implosion and nonlinear oscillations, which enhanced the convective mass transfer between the plant materials and solvent [69,71]. However, the yields exhibited a diminishing trend with ultrasonic time beyond 40 min (50–60 min), which might be attributed to the thermal degradation of heat-sensitive compounds or oxidative damage (caused by prolonged exposure to cavitation-induced reactive oxygen species). Notably, these findings corroborated the research of Hong et al. [72], in which a similar decline was found in polyphenol yield from cinnamon bark with ultrasonication exceeding 30–40 min. Overall, it might be indicated by this phenomenon that an equilibrium establishment existed between the extract and NADES, which was coupled with competing processes, such as co-extraction of interfering matrix components and thermal degradation of target analytes [73]. Based on the comprehensive kinetic evaluation and extraction efficiency assessments, 40 min was identified as the optimal ultrasonic time in this research.

#### 2.3.2. Ultrasonic Temperature

Generally, ultrasonic temperature exhibited significant influences on the solvation efficiency of target phytochemicals, while both compound stability and mass transfer kinetics could be enhanced under the optimal thermal conditions [74]. Figure 4B demonstrates the temperature-dependent extraction profile (20–70 °C) of eight bioactive compounds from HEL, in which all other extraction parameters (ultrasonic time of 40 min, solid/liquid ratio of 1:50 g/mL, ultrasonic power of 400 W) were maintained constant for the specific evaluation of the thermal effects. The total yield of target components exhibited a distinct temperature dependence, which reached the peak at 40 °C (with a 21.519 mg/g enhancement compared to 20 °C), and this enhancement could be attributed to several thermally mediated mechanisms: enhanced molecular mobility within the NADES system that accelerated the diffusion kinetics, improved cellular membrane permeability that facilitated the bioactive constituent transfer, and temperature-dependent reduction in NADES viscosity that enhanced the solid–liquid interfacial contact. Consequently, the mass transfer efficiency could be optimized based on the synergistic effects of these mechanisms. However, the extraction yield exhibited a progressive decline, which could be attributed to the competitive effects between two opposing phenomena: the beneficial effects of temperature on mass transfer kinetics and the negative effects on ultrasonic cavitation efficiency. According to the studies, elevated temperatures could increase the vapor pressure within collapsing bubbles, thereby reducing the cavitation intensity and weakening the mechanical effects (essential for effective cell wall disruption) [75]. Notably, it was revealed by the thermodynamic analysis that 40 °C was the optimal condition to reach a balance between the thermally enhanced mass transfer and ultrasound-mediated extraction efficiency, which could simultaneously maximize the target compound recovery while maintaining molecular stability. Therefore, 40 °C was determined as the optimal ultrasonic temperature in this research.

#### 2.3.3. Ultrasonic Power

Figure 4C illustrates the power-dependent extraction profile of bioactive compounds from HE, in which a progressive increase in extraction efficiency could be observed across the varying ultrasonic power ranging from 160 to 400 W at 40 °C with a solid/liquid ratio of 1:50 g/mL for 40 min. According to the results, the maximum yield was obtained at 400 W, which could represent the optimal balance between cavitation intensity and compound stability for effective matrix disruption and phytochemical solubilization, and the enhanced extraction efficiency could be attributed to two primary mechanisms: ultrasonication-induced cavitation generated localized extreme conditions that created substantial shear forces, which could effectively disrupt the cellular structures and release intracellular compounds; and the enhancement of NADES penetration through these structural defects, which was based on the power-dependent reductions in solvent viscosity and enhanced wettability [76]. However, the extraction yields exhibited a decreasing trend with power beyond 400 W, which could be attributed to three interrelated factors: excessive bubble formation created a shielding effect that reduced the energy transfer efficiency, localized overheating at bubble collapse sites promoted thermal degradation of sensitive compounds, and elevated free radical generation from intensified bubble collapse that induced oxidative damage to target phytochemicals. Notably, these observations were consistent with the findings, which focused on the ultrasonic extraction of curcumin and gingerenone A from *Zingiber officinale* [77]. Based on the comprehensive yield analysis and stability assessment, 400 W was conclusively identified as the optimal ultrasonic power for HE processing, which could realize the maximal extraction efficiency while maintaining compound integrity.

#### 2.3.4. Solid/Liquid Ratio

During the NADES-UAE processes, the solid/liquid ratio represents a critical parameter in balancing extraction yield with solvent economy. The remaining extraction parameters were held constant throughout the experiments, including ultrasonic time (40 min), ultrasonic temperature (40 °C), and ultrasonic power (400 W). As illustrated in Figure 4D, a progressive enhancement in extraction yields for all eight bioactive constituents could be observed in the systematic evaluation across a gradient of solid/liquid ratio (1:10–1:50 g/mL), and it reached the maximum efficiency at 1:50 g/mL. This ratio-dependent behavior could reflect complementary mechanisms: compound-specific solubility thresholds hold a demand for a sufficient solvent volume for the complete dissolution of hydrophobic constituents, alongside the viscosity modulation effects (increased liquid phase proportion will reduce NADES viscosity), which can lower the cavitation thresholds and enhance the mass transfer kinetics based on improved solvent penetration. Notably, competing phenomena occurred with a yield plateau observed beyond 50 g/mL, while increased solvent volume initially promoted the compound dissolution based on enhanced phase contact, and the excessive dilution (>50 g/mL) induced co-extraction of matrix interferents that impeded target compound recovery through competitive solvation effects. Based on the combined consideration of extraction efficiency and solvent sustainability metrics, the solid/liquid ratio of 1:50 g/mL was selected as the optimal condition.

### 2.4. BBD-RSM Results

RSM was employed to systematically optimize the multivariate extraction system, which could effectively resolve the nonlinear interactions between key process parameters [78,79]. In addition, a BBD was implemented (Table 3), in which 17 experimental runs were operated to characterize three critical factors: ultrasonic temperature (X_1_), solid/liquid ratio (X_2_), and ultrasonic time (X_3_). The ranges of the independent variables selected for the RSM study were based on the results of single-factor experiments. The exact ranges were as follows: ultrasonic temperature (X_1_, 30–50 °C), solid/liquid ratio (X_2_, 30–70 g/mL), and ultrasonic time (X_3_, 20–60 min). These ranges were chosen to ensure they encompassed the optimal regions identified in the preliminary single-factor investigations while maintaining operational feasibility. Notably, the resulting second-order polynomial model equation demonstrated strong predictive capability for total extraction yield (Y): Y = 176.33 + 11.49X_1_ + 11.30X_2_ + 5.03X_3_ + 0.4811X_1_X_2_ + 8.06X_1_X_3_ + 2.64X_2_X_3_ − 32.62X_1_^2^ − 27.44X_2_^2^ − 14.07X_3_^2^. In addition, the exceptional statistical significance (*p* < 0.0001) and predictive accuracy (lack of fit: *p* > 0.05) of the model could be further confirmed by ANOVA analysis in Table 4 [80]. According to the parametric sensitivity analysis, the factor hierarchy was as follows: ultrasonic temperature (X_1_) > solid/liquid ratio (X_2_) > ultrasonic time (X_3_), with particularly strong effects (*p* < 0.01) in quadratic terms X_1_^2^, X_2_^2^ and X_3_^2^. It could be indicated by the high correlation coefficient (R^2^ = 0.9778) and adjusted R^2^ (0.9493) that the model accounted for 97.0% of the total variation, alongside the existence of minor unaccounted experimental variables [81].

**Figure 5:**
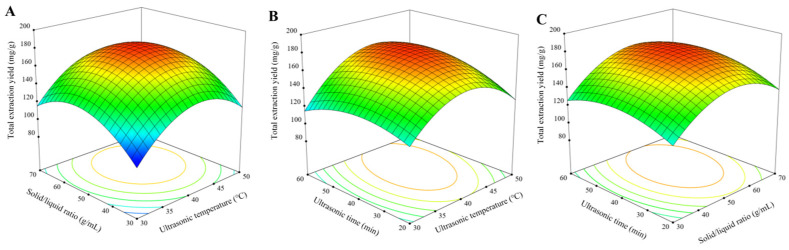
Response surfaces for the total extraction yield (**A**–**C**). A varying ultrasonic temperature and solid/liquid ratio; B varying ultrasonic temperature and ultrasonic time; C varying solid/liquid ratio and ultrasonic time (*n* = 3).

Response surface analysis was employed to visualize the effects of the extraction parameters and their interactions on the total extraction yield, and the results are listed in Figure 5. Specifically, the gradient of the response surface contours provides critical insights into the interaction effects between process variables, alongside the significant factor interactions indicated by the pronounced curvature, whereas minimal topographic variation suggests negligible interactive effects [82]. The three-dimensional response surface plots (Figure 5) revealed distinct topographic variations, with the most significant gradient (*p* < 0.01) in ultrasonic temperature, which could underscore its dominant influence on the total extraction yield. In contrast, ultrasonic time showed minimal surface curvature, which was consistent with its relatively weaker effects (*p* > 0.05) on extraction efficiency. Furthermore, Figure 4A displays the interaction between ultrasonic temperature and solid/liquid ratio on the yield of the eight components. According to the results, the extraction yields of all target components exhibited a parabolic trend with the increase in ultrasonic temperature and solid/liquid ratio, and they reached the maxima under the optimal intermediate conditions. Based on the above findings, it could be indicated that moderate ultrasonic temperature and solid/liquid ratio exhibited positive effects on the total extraction yield (Figure 4A). Subsequently, the total extraction yield exhibited a decreasing trend when the solid/liquid ratio exceeded 1:54 g/mL. Although a higher solid/liquid ratio could increase the solvent-contact area and enhance the solubilization of intracellular components, excessive water content might disrupt hydrogen bonding within the NADES system, resulting in the decline of extraction efficiency [83]. Similarly, the total yield of the eight components exhibited a significant decreasing trend when the ultrasonic temperature exceeded 42 °C. Although the elevated temperatures could accelerate the mass transfer and solubility, excessively high temperatures would degrade the thermolabile active components [71]. In summary, the optimal extraction conditions were determined as a solid/liquid ratio of 1:54 g/mL, an ultrasonic time of 45 min, and an ultrasonic temperature of 42 °C. It was verified by response surface analysis that ultrasonic temperature significantly enhanced yields (*p* < 0.01), whereas the effects of ultrasonic time were relatively negligible (*p* > 0.05), which was in agreement with previous studies [84]. Notably, ultrasonic temperature exhibited a slight correlation with ultrasonic time, in which the temperature exerted a positive effect on extraction efficiency, while time exhibited no significant impact. Figure 4C displayed the interaction between solid/liquid ratio and ultrasonic time, and it was revealed that the highest extraction yield occurred at a solid/liquid ratio of 1:54 g/mL and an ultrasonic time of 49 min.

As predicted by the regression model, the optimal NADES-UAE (Mal-DMU-1,4-BDO) extraction conditions were as follows: 45 min ultrasonic time, a solid/liquid ratio of 1:54 g/mL, and an ultrasonic temperature of 42 °C. Experimental validation was conducted under the aforementioned experimental conditions, and a total extraction yield was 179.431 ± 0.790 mg/g (*n* = 3), which was in excellent agreement with the predicted value of 180.429 mg/g (relative error < 3%). The low SD (±0.790) and minimal relative error collectively confirm the high accuracy and reproducibility of the RSM model. Overall, the predictive accuracy of the BBD-RSM model was confirmed by the high coefficient of determination (R^2^ = 0.9778), adjusted R^2^ (adj-R^2^ = 0.94934), and non-significant lack-of-fit test (*p* = 0.2254), collectively demonstrating the robustness of the model and verifying the efficiency and reliability of the optimized extraction protocol.

### 2.5. Comparative Analysis of UAE and Traditional Extraction Methods

**Figure 6:**
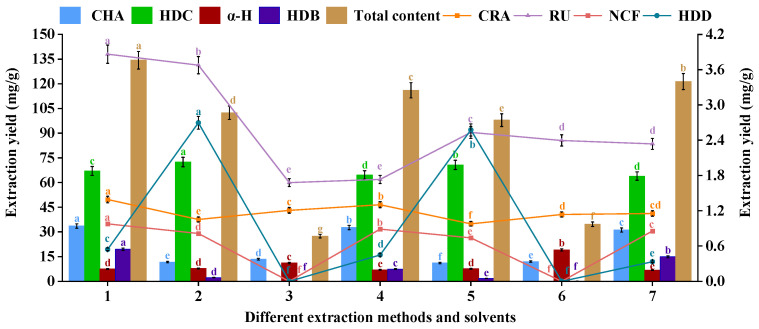
Comparative evaluation of extraction methods and solvents on eight bioactive constituents in *Hedera helix*. Data are presented as mean ± SD (*n* = 3). Different lowercase letters (a–g) indicate statistically significant differences (*p* < 0.05, Tukey’s test).

A systematic comparative analysis with conventional extraction techniques (HRE and CSE) was employed for the extraction of eight bioactive constituents from HE leaves, which aimed to validate the superiority of UAE. According to the results, UAE was the most effective method for the extraction of phenolic, flavonoid, and saponin constituents, and it might be attributed to the prolonged extraction time and elevated temperatures associated with HRE, which could promote the degradation of thermolabile phenolic compounds [85]. In contrast, the UAE realized the rapid temperature elevation based on the cavitation effects, thereby minimizing thermal exposure and preserving the integrity of target constituents. Furthermore, as characterized by extended extraction durations and low efficiency, CSE was surpassed by UAE, which also produced higher-purity extracts [86,87]. It was revealed by comparative analysis that NADES-UAE achieved a significantly higher extraction yield (180.429 mg/g) than NADES-HRE (116.025 mg/g) or NADES-CSE (121.371 mg/g), which might be attributed to the synergistic effects of ultrasound-enhanced mass transfer kinetics and the optimized solvation properties of NADES. According to the comparison of the extraction yields with the application of NADES, MeOH, and water across different extraction methods (Figure 6), NADES consistently outperformed both MeOH and water, with water obtaining the lowest yield of the eight target components. Overall, the NADES-UAE system delivered the highest extraction efficiency among all tested methods, which could highlight the superiority of the NADES-UAE system.

Furthermore, compared with existing methods reported in the literature, the optimized extraction strategy established in this study demonstrates significant advantages in the comprehensive extraction efficiency of multiple active components. For instance, Hussain et al. [46] used 40% ethanol as the extraction solvent, with soaking for 30 min followed by reflux heating at 45 °C for 72 min, achieving a maximum content of HDC of 15.26% in the resulting powdered extract. Although this value is higher than the content obtained in this study (6.704%)-a difference of approximately 8.556%-our approach enabled the efficient and synergistic extraction of the other seven active components from HE. Moreover, the total extraction time was significantly shortened, and the solvent system aligns more closely with green chemistry principles. Overall, the proposed process shows greater potential in terms of both efficiency and comprehensiveness for practical application.

### 2.6. Morphological Changes in HE Before and After Extraction

**Figure 7:**
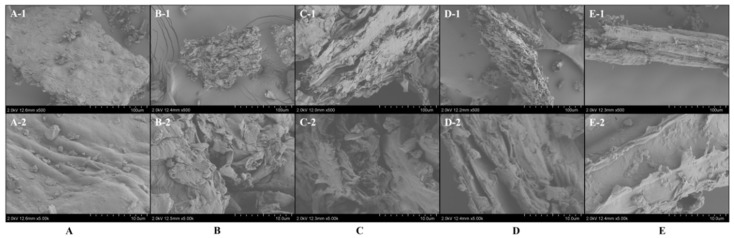
SEM characterization of *Hedera helix* powder: untreated control (**A**), (**A-1**,**A-2**) showing two representative fields of view; NADES-UAE treated (**B**), (**B-1**,**B-2**) showing two representative fields of view; NADES-HRE treated (**C**), (**C-1**,**C-2**) showing two representative fields of view; Methanol-UAE treated (**D**), (**D-1**,**D-2**) showing two representative fields of view; and Water-UAE treated (**E**), showing distinct cell wall disruption patterns correlated with extraction efficiency, (**E-1**,**E-2**) showing two representative fields of view.

According to previous studies, the extraction efficiency of target compounds exhibited an intrinsic connection with the degree of plant cell wall disruption [88,89]. In this study, with the application of different extraction methods (UAE, HRE) and extraction solvents (Mal-DMU-1,4-BDO (1:2:1.5), MeOH, and water), the microstructural changes in HEL were comparatively analyzed by a scanning electron microscope (SU-8010, Hitachi, Japan), to deeply explore the mechanisms underlying solvent- and method-dependent cell wall integrity alterations. The sample preparation involved fixation on the sample stage with double-sided carbon tape, followed by metallization before analysis. According to the results, distinct morphological differences could be found between extraction treatments (Figure 7A). For instance, untreated HEL powder displayed intact cellular morphology with smooth surfaces and residual cytoplasmic contents, while extracted samples exhibited pronounced surface folding, pore formation, and removal of surface particulates, indicating that the characteristic cell wall degradation patterns could be induced by both extraction methods and solvents. Compared with HRE-treated counterparts, Mal-DMU-1,4-BDO (1:2:1.5)-UAE-treated samples demonstrated superior porosity and more extensive surface folding, alongside the particularly prominent flocculent surface morphology. Notably, the enhanced disruption observed in UAE-treated samples could be attributed to two primary mechanisms, ultrasonic cavitation-induced implosion bubbles that mechanically eroded the powder surface, and high-frequency vibrations that promoted superior solvent penetration into cellular matrices [59,90]. These effects synergistically resulted in structural shrinkage and exacerbation of pore rupture on cell surfaces, which further facilitated the content release. Moreover, it could be further revealed by the microstructural analysis (Figure 7B,D,E) that Mal-DMU-1,4-BDO (1:2:1.5)-extracted samples displayed significantly greater porosity and distinctive flocculent morphology versus MeOH- or water-extracted samples. It could be suggested that the selective interaction of Mal-DMU-1,4-BDO (1:2:1.5) with cellulose components could promote more efficient cell wall dissolution [91], thereby enhancing the release and solubilization of intracellular bioactive constituents, ultimately contributing to the extraction yields.

### 2.7. Effects of NADES on HT-29 Colon Cancer Cells

**Figure 8:**
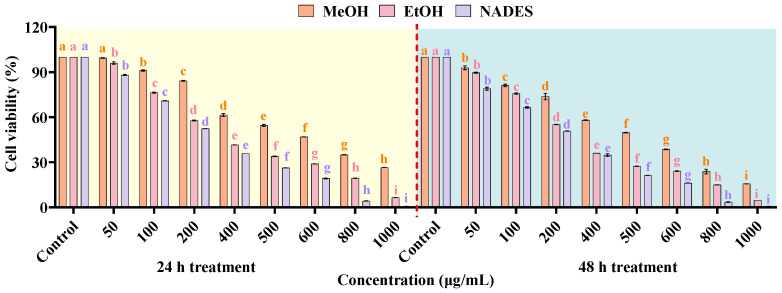
Comparative cytotoxicity of solvent extracts on HT-29 cell viability assessed by CCK-8 assay: 24 h and 48 h treatment (*n* = 3), different lowercase letters (a–i) indicate statistically significant differences (*p* < 0.05, Tukey’s test).

The analysis of the cytotoxic activity was conducted using HT-29 human colorectal adenocarcinoma cells, which were treated with extracts obtained from Mal:DU:1,4-BDO (1:2:1.5), MeOH, and EtOH extraction systems, in order to comparatively evaluate the extraction efficacy of NADES versus conventional organic solvents. In addition, cell viability was quantitatively analyzed at standardized intervals (24 h and 48 h) post-treatment based on the CCK-8 assay. Specifically, the extracts were prepared at concentrations ranging from 50 to 1000 μg/mL, and the samples were compared with the controls. As shown in Figure 8, all three extraction solvents exhibited significant cytotoxic effects on HT-29 cells in a dose- and time-dependent manner. Notably, the extracts demonstrated a marked effect even at the lowest concentration of 50 μg/mL (*p* ≤ 0.001), and the highest cytotoxicity was observed at 1000 μg/mL. In addition, the antiproliferative activity of the extracts exhibited an enhanced trend over time, which could suggest a progressive accumulation of bioactive constituents within cells. This observed cytotoxicity could be attributed to the action mechanisms of the bioactive ingredients in HE extracts. For instance, the saponin constituents have been reported to induce apoptosis based on the modulation of key oncogenic pathways (e.g., PI3K/Akt and NF-κB), alongside the disruption of the redox balance in cancer cells [92,93]. In addition, phenolic constituents (such as CHA) exhibited significant anticancer activity by triggering mitochondrial/caspase-dependent apoptosis [94]. Additionally, flavonoids (such as RU) could further enhance this effect by upregulating the pro-apoptotic Bax proteins while downregulating the expression of anti-apoptotic Bcl-2, thereby promoting the process of PARP cleavage [95]. Overall, these findings underscore the ability of HE extracts to impede colon cancer cell proliferation, highlighting their potential application as therapeutic agents.

Compared with MeOH- and EtOH-derived extracts, NADES-extracted HEL exhibited superior cytotoxic activity against HT-29 cells across all concentrations and time points (Figure 8), which might be attributed to the unique physicochemical properties of the NADES formulation, contributing to a characteristically low pH value. Given that tumor microenvironments tended to exhibit acidic extracellular pH while maintaining neutral intracellular pH based on robust proton gradient regulation, Mal might disrupt this equilibrium, thus inducing intracellular acidification and subsequent cancer cell death [96]. Furthermore, it was confirmed by HPLC analysis that higher concentrations of bioactive constituents could be extracted by the Mal:DMU:1,4-BDO (1:2:1.5) system, which might be attributed to the pronounced antitumor activity.

**Figure 9:**
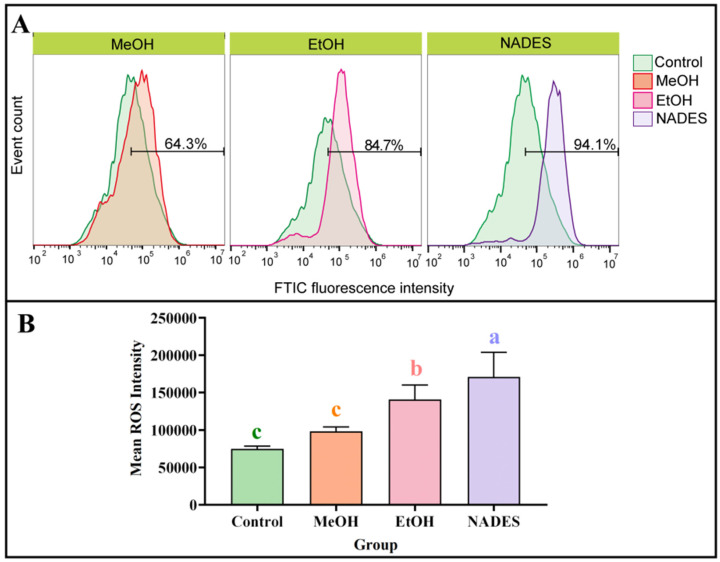
Comparative analysis of ROS generation in HT-29 cells treated with different solvent extracts: Representative flow cytometry histograms (**A**) and Quantitative comparison of ROS levels (**B**) (*n* = 3); different lowercase letters (a–c) indicate statistically significant differences (*p* < 0.05, Tukey’s test).

In this research, the inhibition of HT-29 cell proliferation and induction of mitochondrial autophagy were both found to exhibit a strong connection with oxidative stress, which manifested as alterations in intracellular ROS levels. The ROS production in HT-29 cells was treated with three HEL extracts at an equivalent concentration (50 μg/mL) through flow cytometry, in order to further investigate the antiproliferative effects of different extracts (obtained from different solvent systems), followed by fluorescence intensity analysis based on FlowJo software 10.9. Compared with the blank group, ROS levels were significantly elevated in the control group (Figure 9). Notably, all three solvent extracts (MeOH, EtOH, and Mal:DMU:1,4-BDO) substantially increased intracellular ROS levels in HT-29 cells, which could suggest the ability of HEL extracts to promote apoptosis through ROS-mediated pathways. These findings are consistent with the results of CCK-8 assays, and it is noteworthy that the three extraction solvents exhibit a strong connection with the superior cytotoxic activity in previous experiments. Overall, the strongest inhibitory effects of NADES-derived extracts on HT-29 cell proliferation could be further confirmed by these results.

## 3. Materials and Methods

### 3.1. Plant Material

Fresh HEL were collected from Songyang District, Lishui City, Zhejiang Province, China (28°14′ N, 119°10′ E), on 3 May 2024. The plant material consisted of six-month-old cultivated ivy, with mature leaves collected from the basal growth region and was authenticated by Assistant Researcher Hongliang Chen. A voucher specimen (ZCMU-HH-20240503-01) has been deposited in the Herbarium of Zhejiang Chinese Medical University. The collected HEL was rinsed with deionized water, dried at 50 °C for 12 h, yielding an initial moisture content of 33.385% (Relative Standard Deviation, RSD ≤ 1.986%), subsequently ground into powder by a high-speed grinder, sieved through a 50-mesh sieve, and stored in airtight containers.

### 3.2. Chemicals and Reagents

All chemical reagents and biological materials in this study were directly purchased from internationally recognized suppliers. Choline chloride (ChCl, 99%), betaine (Bet, 98%), urea (Ur, 98%), acetamide (Am, 99%), citric acid (Ca, 99.5%), lactic acid (Lac, 85%), 1,2-propanediol (1,2-Pg, 99%), 1,4-butanediol (1,4-BDO, 99%), ethylene glycol (EG, 99%), N,N′-dimethylurea (DMU, 98%), and malonic acid (Mal, 99.5%) were obtained from Shanghai McLean Biochemical Technology Co., Ltd. (Shanghai, China). Standards for bioactive compounds, including CHA, CRA, RU, NCF, HDC, HDB, HDD, and α-H (all ≥98% purity), were supplied by Shanghai Yuan Ye Biotechnology Co., Ltd. (Shanghai, China). In addition, HPLC-grade solvents (including MeOH, EtOH, and acetonitrile) were acquired from Sigma-Aldrich (St. Louis, MO, USA). Cell culture reagents, such as fetal bovine serum (FBS), phosphate-buffered saline (PBS), CCK-8, and culture medium, were purchased from White Shark Biotechnology Co., Ltd. (Shanghai, China). Additionally, the reactive oxygen species (ROS) detection kit was obtained from Shanghai Biyuntian Biotechnology Co., Ltd. (Shanghai, China).

### 3.3. Extraction of NADES-UAE

The NADES systems were prepared according to the compositions in Table S1. HBA and the respective HBD at a predetermined molar ratio in a round-bottom flask, with the addition of 30% (*v*/*v*) deionized water as a cosolvent. Subsequently, the mixture was magnetically stirred at 100 rpm under controlled heating (80 °C) until fully homogenized, following standard procedures for NADES preparation [22,23]. Based on optimized conditions reported for similar plant matrices, for bioactive compound extraction, 1.0 g of dried HEL powder was precisely weighed and mixed with 50 mL of the optimized NADES in an ultrasonic bath at 50 °C for 40 min [21]. Ultrasonic treatment in this study was performed using a benchtop ultrasonic cleaner (model SB25-12DTD, Ningbo Scientz Biotechnology Co., Ltd., Ningbo, China) equipped with a 22.5 L chamber (50 cm × 30 cm × 15 cm), operating at a fixed frequency of 40 kHz and delivering a maximum power output of 600 W, yielding an ultrasonic power density of 0.0267 W/cm^3^ for uniform energy distribution. The system allowed precise temperature control (25–80 °C) and automated duration adjustment (1–999 min). This high-performance ultrasonic system ensured consistent and uniform sonication throughout all experimental procedures. Prior to HPLC injection, the extraction mixture was centrifuged at 8000× *g* at 4 °C for 10 min, and the resulting supernatant was filtered through a 0.22 μm nylon membrane without dilution. The obtained particle-free filtrate was directly injected into the HPLC system for analysis.

### 3.4. Optimization of the Extraction Process

#### 3.4.1. Single-Factor Experiment

Based on a comprehensive review of existing literature and preliminary experimental validation, 15 NADES formulations were selected. Subsequently, a systematic screening of these formulations was conducted, with varying component ratios in each of them, which aimed at the construction of an optimal green extraction system. Additionally, to ensure the reproducibility and consistency of NADES properties, ChCl was rigorously dried under vacuum at 60 °C for 24 h before use. With the application of this optimized NADES system (Section 3.2), the target constituents from HEL were systematically extracted and analyzed to evaluate the extraction efficiency for eight bioactive compounds. Additionally, based on preliminary studies on NADES-UAE from medicinal plants, the effects of various parameters on the extraction process were systematically evaluated through single-factor experiment. These experiments investigated the composition of NADES, molar ratio, water content (10–90%), and ultrasound parameters including ultrasonic time (10–70 min), temperature (20–70 °C), power (160–600 W), and solid/liquid ratio (1:10–1:60 g/mL) to establish the optimal ranges for subsequent multivariate optimization [39]. In addition, these parameters were then investigated according to the method described in Section 3.3.

#### 3.4.2. Experimental Design

Based on established methodologies for response surface methodology optimization in plant extraction studies, 17 RSM experiments with a BBD were employed to investigate the interaction effects of solid/liquid ratio, ultrasonic time, and ultrasonic temperature on eight HE components, in which second-order polynomial equations were developed to model the responses, and the variable relationships were analyzed by ANOVA (*p* < 0.05) using Design-Expert 13 software (Stat-Ease, Inc., Minneapolis, MN, USA) [32]. In addition, in our experimental design, a total of five center points (runs 13–17) were incorporated to estimate pure error and assess model curvature.

### 3.5. Determination of HE Content by HPLC

A validated HPLC-DAD method was employed to quantitatively analyze the bioactive constituents in HEL extract (including CHA, CRA, RU, NCF, HDC, HDD, HDB, and α-H), and this analysis was based on an Agilent 1260 Infinity II system (Agilent Technologies, Santa Clara, CA, USA) equipped with a quaternary pump, autosampler, and diode array detector. A Horizon C18 column (250 × 4.6 mm, 5 μm) was employed for chromatographic separation, and the temperature was maintained at 30 °C. The mobile phase consisted of 0.2% aqueous phosphoric acid (A) and acetonitrile (B), with the following gradient program: initial 5–12% B (0–6 min), 12–22% B (6–19 min), 22–24% B (19–25 min), 24–32% B (25–31 min), 32–33% B (31–40 min), maintained at 33–50% B (40–44 min), followed by a rapid increase to 54% B (44–45 min), then decreased to 100% B (45–55 min), and finally returned to initial conditions (55–60 min). The flow rate was maintained at 1.0 mL/min with a 10 μL injection volume, and detection was performed at 205 nm. Compound identification was established based on the comparison of retention times and UV spectra (200–400 nm) with authentic standards. Quantification was performed by external calibration curves (R^2^ > 0.999), which were generated from serial dilutions of reference standards. OpenLab CDS software (v3.6, Agilent Technologies, Santa Clara, CA, USA) was employed to process all chromatographic data, and the results were expressed as mg compound per g dry weight (mg/g DW).

### 3.6. Evaluation of Anti-Colon Cancer Capacity

#### 3.6.1. Cell Culture

Under standardized conditions, the human colorectal adenocarcinoma HT-29 cell line was cultured in DMEM (supplemented with 10% heat-inactivated FBS), and the samples were maintained at 37 °C in a humidified incubator with a 5% CO_2_ atmosphere [97,98]. Additionally, cell monolayers were routinely monitored and subcultured by 0.25% trypsin-EDTA solution upon reaching 85–90% confluency, which aimed at cell detachment. After the enzymatic dissociation, cells were resuspended in a complete medium and seeded at appropriate densities in either multi-well plates or Petri dishes, following the specific experimental requirements.

#### 3.6.2. HE Reserve

The bioactive compounds from HEL were systematically extracted by three distinct solvent systems (as detailed in Section 3.3): conventional organic solvents (MeOH and EtOH) and an optimized NADES. After the centrifugation, the resulting supernatants were concentrated under reduced pressure at 60 °C by a rotary evaporator to remove the solvents (MeOH, EtOH and NADES), reconstituted with an appropriate volume of deionized water to obtain the aqueous extract, and subsequently lyophilized to yield solvent-free extracts [99]. To ensure the precise dosing in subsequent bioassays, a primary stock solution (10 mg/mL) was prepared through the dissolution of 10.0 mg of extract in 10 mL of medium, and the hydrophilic extracts were standardized by the primary stock solution. Hydrophobic extracts were dispersed directly in cell culture medium via ultrasonication (40 kHz, 200 W, 30 min, 37 °C) without solubilizing agents. Homogeneity was ensured by vortex mixing every 10 min and confirmed by the visual absence of precipitation. Fresh suspensions were used within 30 min of preparation. This solution was subsequently sterile-filtered through a 0.22 μm membrane and stored at −20 °C, and the samples would be aliquots thawed immediately before the experiments to prevent compound degradation.

#### 3.6.3. Cell Viability

This study investigated the concentration- and time-dependent inhibitory effects of HEL extracts on human colorectal adenocarcinoma HT-29 cells through a standardized CCK-8 assay, in which the HEL extracts were prepared using methanol, ethanol, and optimized NADES extraction methods. Specifically, HT-29 cells were seeded in 96-well plates at 1 × 10^4^ cells/well and cultured overnight under standard conditions (37 °C, 5% CO_2_) before treatment, followed by disposing with serially diluted HEL extracts (0–1000 μg/mL) for 24 and 48 h, with culture medium-only and solvent-matched vehicle controls [99]. After the treatment mentioned above, cells were washed with PBS and incubated with CCK-8 reagent (10% *v*/*v*) for 60 min, followed by measurement of the absorbance at 450 nm by a Synergy H1 microplate reader with 650 nm reference correction. Cell viability was calculated relative to untreated controls, and all experiments were performed in six replicates to ensure statistical robustness. Cell viability was calculated as: Viability (%) = [(A_sample_ − A_blank_)/(A_negative control_ − A_blank_)] × 100.

#### 3.6.4. Reactive Oxygen Species (ROS)

Intracellular ROS levels were determined using the fluorescent probe 2′,7′-dichlorodihydrofluorescein diacetate (DCFH-DA) through a standardized protocol [100]. The cell-permeable DCFH-DA probe underwent intracellular hydrolysis by endogenous esterases to form non-fluorescent DCFH, which was subsequently oxidized by ROS to yield highly fluorescent 2′,7′-dichlorofluorescein (DCF). Subsequently, cells were incubated with 10 μM DCFH-DA working solution in a complete culture medium at 37 °C for 30 min under a 5% CO_2_ atmosphere. After the incubation, cells were immediately washed with ice-cold PBS to terminate the reaction, followed by maintaining on ice to preserve the oxidative state. DCF fluorescence intensity was proportional to intracellular ROS levels, and it was quantified by flow cytometry (excitation/emission: 488/525 nm) within 30 min of staining, to ensure the measurement accuracy.

### 3.7. Comparison of Different Extraction Methods and Solvents

This study aimed at the identification of the optimal extraction method and solvents for the determination of the content of extracts from HEL. Specifically, the methods were composed of UAE, HRE, CSE, and extraction solvents using MeOH, EtOH, and NADES (Table 5).

### 3.8. Statistical Analysis

All experimental data were acquired by three independent biological replicates and expressed as mean values ± standard deviation (SD). For the optimization experiments using RSM, Design-Expert^®^ software (Version 13, Stat-Ease Inc., Minneapolis, MN, USA) was employed to generate the experimental design, fit a second-order polynomial model, and perform the corresponding analysis of variance (ANOVA). Type III sum of squares was used, and model terms were considered statistically significant at *p* < 0.05. The software’s automatic model reduction feature was applied to maintain hierarchy while removing non-significant terms (*p* > 0.05). For other comparative analysis, statistical significance was determined by one-way analysis of variance (ANOVA), followed by Tukey’s post hoc test using IBM SPSS Statistics software (version 27; IBM Corp., Armonk, NY, USA). OriginPro 2024 (OriginLab Corporation, Northampton, MA, USA) and GraphPad Prism 9.6.0 (GraphPad Software, San Diego, CA, USA) were employed for the data visualization and advanced statistical analyses. The threshold for statistical significance was set at *p* < 0.05 for all comparisons.

## 4. Conclusions

A novel NADES-UAE approach was employed in this study, which could serve as an alternative to conventional extraction methods for the extraction of eight bioactive ingredients from HEL. Based on RSM optimization, the optimal extraction parameters were determined as: Mal-DMU-1,4-BDO (1:2:1.5, molar ratio) as the NADES, 30% water content, a solid/liquid ratio of 1:54 g/mL, ultrasonic temperature of 42 °C, ultrasonic time of 45 min, and ultrasonic power of 400 W. Under these optimized conditions, 180.429 mg/g of the target components could be yielded. The hydrogen bonding interactions among the components of NADES could be confirmed by the FT-IR analysis, viscosity comparison, alongside the clarification of the molecular mechanisms. In addition, it was revealed by comparative analysis via SEM that the NADES-UAE system resulted in significantly more structural damage to HEL powder versus other solvents and methods, which could verify its superior extraction efficiency. The enhanced bioactivity of NADES-derived extracts was further validated by CCK-8 assays, which demonstrated optimal inhibition of HT-29 cell proliferation. Moreover, it was indicated by the flow cytometric analysis that NADES-UAE extracts induced oxidative stress in HT-29 cells through significant elevation of intracellular ROS, which significantly promoted cancer cell apoptosis.

Compared to conventional extraction methods for HEL, this study integrates the emerging NADES-UAE technique with HEL, representing a significant advancement in sustainable bioprocessing. This eco-friendly approach reinforces circular economy principles, enhances process efficiency and selectivity, and increases product yield, thereby facilitating its implementation in various industrial sustainability initiatives. However, this research faces challenges in scaling up production and ensuring economic viability. Future studies should focus on optimizing process parameters for large-scale extraction and expanding the applicability of NADES-UAE to diverse production scenarios and waste stream treatment. By addressing these challenges, the NADES-UAE approach can effectively bridge the gap between academic innovation and industrial application, creating a sustainable pathway for developing high-value, biobased products and ultimately promoting HE as an emerging economic crop.

## Figures and Tables

**Table 1:** Method validation parameters for simultaneous quantification of eight bioactive compounds in *Hedera helix* leaves (*n* = 6).

Analyte	Linearity Range (μg/mL)	Retention Time (min)	LOD (μg/mL)	LOQ (μg/mL)	Intra-Day RSD		Inter-Day RSD		RSD		Recovery (%)	RSD (%)
					Pa (%)	Rt (%)	Pa (%)	Rt (%)	Pa (%)	Rt (%)		
CHA	31.25–1000	12.873	0.060	0.150	0.443	0.055	0.459	0.656	0.691	0.096	99.183	2.337
CRA	31.25–1000	13.689	0.089	0.313	0.647	0.047	1.201	0.163	1.363	0.061	101.589	3.355
RU	62.5–1000	20.725	0.024	0.086	0.326	0.041	0.020	0.081	0.045	0.032	100.916	4.735
NCF	31.25–1000	23.405	0.014	0.047	0.418	0.026	0.375	0.050	1.985	0.266	99.889	0.392
HDC	187.5–12,000	35.539	0.560	1.900	0.320	0.984	0.227	0.022	1.228	0.021	97.956	0.374
HDD	15.625–2000	36.309	0.109	0.360	0.442	0.014	0.300	0.024	1.570	0.018	102.783	0.941
HDB	50–1600	44.473	0.820	2.900	0.807	0.013	0.512	0.019	1.570	0.017	99.500	3.239
α-H	187.5–12,000	49.800	0.516	1.550	0.293	0.008	0.254	0.010	1.570	0.010	100.211	1.179

**Table 2:** Viscosity of NADES (Mal:DMU:1,4-BDO) formulations with different molar ratios at 25 °C (*n* = 3).

No	NADES Formulation (HBA:HBD-1:HBD-2)	Molar Ratio (HBA:HBD-1:HBD-2)	Viscosity (mPa·s)
1	Mal:DMU:1,4-BDO	1:2:1	16.640 ± 0.001
2		1:2:1.5	18.464 ± 0.088
3		1:2:2	18.816 ± 0.088
4		1:2:2.5	19.008 ± 0.072
5		1:2:3	19.680 ± 0.001
6		1:2:3.5	19.744 ± 0.088
7		1:2:4	19.840 ± 0.001
8		1:2:5	21.472 ± 0.072
9		1:2:6	21.504 ± 0.001
10		1:2:7	21.600 ± 0.072

**Table 3:** Results of the BBD for the total extraction yield (*n* = 3).

Runs	Factors			Total Extraction Yield (mg/g DW)
	X_1_ (Temp ^a^, °C)	X_2_ (L ^b^, g/mL)	X_3_ (t ^c^, min)	
1	−1(30)	−1(30)	0(40)	98.379 ± 0.810
2	1(50)	−1(30)	0(40)	116.556 ± 1.000
3	−1(30)	1(70)	0(40)	115.011 ± 0.661
4	1(50)	1(70)	0(40)	135.113 ± 0.999
5	−1(30)	0(50)	−1(20)	122.613 ± 1.003
6	1(50)	0(50)	−1(20)	133.312 ± 0.251
7	−1(30)	0(50)	1(60)	109.829 ± 1.002
8	1(50)	0(50)	1(60)	152.779 ± 1.001
9	0(40)	−1(30)	−1(20)	115.261 ± 0.998
10	0(40)	1(70)	−1(20)	137.607 ± 0.999
11	0(40)	−1(30)	1(60)	126.744 ± 0.998
12	0(40)	1(70)	1(60)	159.642 ± 0.996
13	0(40)	0(50)	0(40)	169.626 ± 0.999
14	0(40)	0(50)	0(40)	181.892 ± 1.001
15	0(40)	0(50)	0(40)	174.246 ± 1.001
16	0(40)	0(50)	0(40)	175.162 ± 0.999
17	0(40)	0(50)	0(40)	180.721 ± 1.002

^a^ Ultrasonic temperature (°C), ^b^ Solid/liquid ratio (g/mL), ^c^ Ultrasonic time (min).

**Table 4:** ANOVA statistics of the model for the total extraction yield.

Variables	Mean Square	*F*-Value	*p*-Value	
Model	1324.92	34.26	<0.0001	significant
X_1_	1056.36	27.32	0.0012	
X_2_	1022.29	26.44	0.0013	
X_3_	202.03	5.22	0.0562	
X_1_^2^	4481.00	115.88	<0.0001	
X_2_^2^	3170.84	82.00	<0.0001	
X_3_^2^	833.98	21.57	0.0024	
X_1_X_2_	0.9258	0.0239	0.8814	
X_1_X_3_	260.04	6.72	0.0358	
X_2_X_3_	27.84	0.7198	0.4243	
Lack of fit	56.61	2.24	0.2254	not significant
R^2^	0.9778			
Adjusted R^2^	0.9493			

**Table 5:** Optimization of extraction parameters using different solvents and extraction methods.

No.	Method	Solvent Composition (mol/mol)	Temperature (°C)	Solid/Liquid Ratio (g/mL)	Ultrasonic Power (W)	Time (min)
1	UAE	Mal:DMU:1,4-BDO = 1:2:1.5	42	54	400	45
2		MeOH	42	54	400	45
3		Water	42	54	400	45
4	HRE	Mal:DMU:1,4-BDO = 1:2:1.5	42	54	400	45
5		MeOH	42	54	-	45
6		Water	42	54	-	45
7	CSE	Mal:DMU:1,4-BDO = 1:2:1.5	RT *	54	-	45

* RT = Room Temperature (typically 25 ± 2 °C).

## Data Availability

The original contributions presented in the study are included in the article/Supplementary Materials. Further inquiries can be directed to the corresponding authors.

## Supplementary Materials

The following supporting information can be downloaded at: https://www.mdpi.com/article/10.3390/ijms27052295/s1.

## Abbreviations

The following abbreviations are used in this manuscript:

α-H	α-Hederin
Am	Acetamide
BBD	Box–Behnken Design
Bet	Betaine
1,4-BDO	1,4-Butanediol
Ca	Citric acid
CCK-8	Cell Counting Kit-8
CHA	Chlorogenic acid
ChCl	Choline Chloride
CRA	Cryptochlorogenic acid
CSE	Cold Soaking Extraction
DESs	Deep Eutectic Solvents
DMU	N,N′-Dimethylurea
EG	Ethylene Glycol
FBS	Fetal Bovine Serum
FT-IR	Fourier-transform infrared Spectroscopy
HBA	Hydrogen Bond Acceptor
HBD	Hydrogen Bond Donor
HDB	Hederacoside B
HDC	Hederacoside C
HDD	Hederacoside D
HE	*Hedera helix*
HEL	*Hedera helix* leaves
HRE	Heating Reflux Extraction
Lac	Lactic Acid
MAE	Microwave-Assisted Extraction
Mal	Malonic Acid
NADES	Natural Deep Eutectic Solvent
NCF	Nicotiflorin
PBS	Phosphate-buffered Saline
1,2-Pg	Propylene Glycol
Pro	L-Proline
ROS	Reactive Oxygen Species
RSD	Relative Standard Deviation
RSM	Response Surface Methodology
RU	Rutin
SD	Standard Deviation
SEM	Scanning Electron Microscope
SFE	Supercritical Fluid Extraction
TDES	Ternary Deep Eutectic Solvent
UAE	Ultrasound-assisted Extraction
Ur	Urea

## References

[1] Green A.F., Ramsey T.S., Ramsey J. (2011). Phylogeny and biogeography of ivies (*Hedera* spp., Araliaceae), a polyploid complex of woody vines. Syst. Bot..

[2] European Directorate for the Quality of Medicines & Health Care (2013). European Pharmacopoeia.

[3] Jin F., Jiang X., Ni X., Yu S., Wu F., Shi X., Mao D., Wang H., Shi Q., Liu Y. (2024). Alpha-Hederin induces incomplete autophagic injury in non-small cell lung cancer by interfering with the lysosomal acidification. Sci. Rep..

[4] Tatia R., Tarcomnicu L., Moldovan Z., Raiciu A.D., Moldovan L., Zalaru C.M. (2023). In vitro antiproliferative activity of triterpenoid saponins from leaves of *Hedera helix* L. grown in Romania. S. Afr. J. Bot..

[5] Mendel M., Chłopecka M., Dziekan N., Wiechetek M. (2011). The effect of the whole extract of common ivy (*Hedera helix*) leaves and selected active substances on the motoric activity of rat isolated stomach strips. J. Ethnopharmacol..

[6] Shokry A.A., El-Shiekh R.A., Kamel G., Bakr A.F., Sabry D., Ramadan A. (2022). Anti-arthritic activity of the flavonoids fraction of ivy leaves (*Hedera helix* L.) standardized extract in adjuvant induced arthritis model in rats in relation to its metabolite profile using LC/MS. Biomed. Pharmacother..

[7] Ahchouch H., Al-Moubaraki A.H., Noor E.A., Hadfi A., Driouiche A., Bammou L., Belkhaouda M.H., Salghi R., Chafiq M., Chaouiki A. (2024). From nature to protection: Unleashing the protective potential of *Hedera helix* leaves against corrosion in harsh acidic environments using experimental and theoretical insights. Arab. J. Chem..

[8] Zdarta A., Smułek W., Pacholak A., Kaczorek E. (2019). Environmental aspects of the use of *Hedera helix* extract in bioremediation process. Microorganisms.

[9] Rozovskii A.Y., Lin G.I. (2003). Fundamentals of methanol synthesis and decomposition. Top. Catal..

[10] Hashemi B., Shiri F., Švec F., Nováková L. (2022). Green solvents and approaches recently applied for extraction of natural bioactive compounds. TrAC Trends Anal. Chem..

[11] Kumar M., Barbhai M.D., Puranik S., Natta S., Senapathy M., Dhumal S., Singh S., Kumar S., Deshmukh V.P., Anitha T. (2023). Combination of green extraction techniques and smart solvents for bioactives recovery. TrAC Trends Anal. Chem..

[12] Choi Y.H., van Spronsen J., Dai Y., Verberne M., Hollmann F., Arends I.W.C.E., Witkamp G.J., Verpoorte R. (2011). Are natural deep eutectic solvents the missing link in understanding cellular metabolism and physiology?. Plant Physiol..

[13] Hikmawanti N.P.E., Ramadon D., Jantan I., Mun’im A. (2021). Natural deep eutectic solvents (NADES): Phytochemical extraction performance enhancer for pharmaceutical and nutraceutical product development. Plants.

[14] Dai Y., Witkamp G.J., Verpoorte R., Choi Y.H. (2013). Natural deep eutectic solvents as a new extraction media for phenolic metabolites in *Carthamus tinctorius* L. Anal. Chem..

[15] Dai Y., Varypataki E.M., Golovina E.A., Jiskoot W., Witkamp G.J., Choi Y.H., Verpoorte R. (2021). Natural deep eutectic solvents in plants and plant cells: In vitro evidence for their possible functions. Adv. Bot. Res..

[16] Li M., Rao C., Ye X., Wang M., Yang B., Wang C., Guo L., Xong Y., Cui X. (2023). Applications for natural deep eutectic solvents in Chinese herbal medicines. Front. Pharmacol..

[17] Wu K., Ren J., Wang Q., Nuerjiang M., Xia X., Bian C. (2022). Research progress on the preparation and action mechanism of natural deep eutectic solvents and their application in food. Foods.

[18] Tu Y., Li L., Fan W., Liu L., Wang Z., Yang L. (2022). Development of green and efficient extraction of bioactive ginsenosides from *Panax ginseng* with deep eutectic solvents. Molecules.

[19] Suresh P.S., Singh P.P., Sharma M., Sharma U. (2023). Multicomponent natural deep eutectic solvents: Super solvents for the efficient extraction of steviol glycosides (rebaudioside A) from *Stevia rebaudiana*. J. Clean. Prod..

[20] Zhang H., Li X.P., Kang M., Li Z., Wang X., Xu J., Han J. (2023). Sustainable ultrasound-assisted extraction of *Polygonatum sibiricum* saponins using ionic strength-responsive natural deep eutectic solvents. Ultrason. Sonochem..

[21] Yang G.Y., Song J.N., Chang Y.Q., Wang L., Zheng Y.G., Zhang D., Guo L. (2021). Natural deep eutectic solvents for the extraction of bioactive steroidal saponins from *Dioscoreae nipponicae* rhizoma. Molecules.

[22] Pan J., Ni Z.J., Thakur K., Khan M.R., Zhang J.G., Wei Z.J. (2025). Bioactivity and application potential of O/W emulsions derived from carboxylic acid-based NADES-extracted total saponins from *Polygonatum cyrtonema* Hua. Food Chem..

[23] Wu H.B., Zhang G.Y., Zhang Y., Guo P.X., Wu H.B., Gao R., Liu T.T. (2025). Natural deep eutectic solvent (NADES)-aided extraction of bioactive compounds from cotton byproducts for agricultural applications: Extraction optimization, structural identification, and bioactivity evaluation. Ind. Crops Prod..

[24] Wang Z., Wang S., Chen Y., Liang L., Yang L., Zeng L. (2025). Ultrasound-assisted betaine-based natural deep eutectic solvents for green extraction of total phenols and flavonoids from *Lithocarpus litseifolius*: Mechanistic insights and anti-hyperuricemic applications. Food Res. Int..

[25] Singh P.P., Anmol, Suresh P.S., Sharma U. (2024). NADES extraction, UHPLC-ELSD-based quantification, and network pharmacology-guided target identification of fourteen specialised metabolites from *Trillium govanianum* Wall. ex D.Don. Phytochem. Anal..

[26] Liu Y., Friesen J.B., McAlpine J.B., Lankin D.C., Chen S.N., Pauli G.F. (2018). Natural deep eutectic solvents: Properties, applications, and perspectives. J. Nat. Prod..

[27] Tang M., Cao J., Wu Z., Ding Y., Ye Q., Li J. (2024). Separation of ginsenosides from *Panax notoginseng* intensified with ionic liquids. Ind. Crops Prod..

[28] Tian B., Qiao Y., Liu Q., Li D., Tian Y. (2017). Structural features and thermal degradation behaviors of extracts obtained by heat reflux extraction of low rank coals with cyclohexanone. J. Anal. Appl. Pyrolysis.

[29] Yu S.T., Han B., Bai X.Y., Liu S.C., Xing X., Zhao D.Q., Liu M.C., Wang S.M. (2020). The cold-soaking extract of Chinese yam (*Dioscorea opposita* Thunb.) protects against erectile dysfunction by ameliorating testicular function in hydrocortisone-induced KDS-Yang rats and in oxidatively damaged TM3 cells. J. Ethnopharmacol..

[30] Khotchai W., Therdthai N., Ritthiruangdej P. (2025). Effect of microwave-assisted extraction on quality and taste profiles of crude extracts from split gill mushroom. J. Agric. Food Res..

[31] Mora J.J., Tavares H.M., Curbelo R., Dellacassa E., Cassel E., Apel M.A., von Poser G.L., Vargas R.M.F. (2025). Supercritical fluid extraction of coumarins and flavonoids from citrus peel. J. Supercrit. Fluids.

[32] Zhang W.T., Duan W., Huang G.L., Huang H.L. (2023). Ultrasonic-assisted extraction, analysis and properties of mung bean peel polysaccharide. Ultrason. Sonochem..

[33] Kumar K., Srivastav S., Sharanagat V.S. (2021). Ultrasound assisted extraction (UAE) of bioactive compounds from fruit and vegetable processing by-products: A review. Ultrason. Sonochem..

[34] Meng Y., Yang H., Li Z., Zhang W., Guo L., Zhang Y., Jiang Y. (2025). Intelligent transformation of ultrasound-assisted novel solvent extraction plant active ingredients: Tools for machine learning and deep learning. Food Chem..

[35] Huang Z., Zhao Y., Yang W., Lang L., Sheng J., Tian Y., Gao X. (2025). Preparation of flavonoids from *Amomum tsaoko* and evaluation of their antioxidant and α-glucosidase inhibitory activities. Food Chem. X.

[36] Zeb L., Shafiq M., Jordheim M. (2025). Advancements in sustainable extraction of seaweed phenolics: Integrating extraction technologies with natural deep eutectic solvents (NADES). Food Chem..

[37] Dzah C.S., Duan Y., Zhang H., Wen C., Zhang J., Chen G., Ma H. (2020). The effects of ultrasound assisted extraction on yield, antioxidant, anticancer and antimicrobial activity of polyphenol extracts: A review. Food Biosci..

[38] Ahmad I., Hikmawan B.D., Maharani D.F., Nisrina N., Arifianti A.E., Mun’im A. (2023). Natural deep eutectic solvent based ultrasound-assisted extraction: A green approach for extraction of sulfhydryl and mimosine from *Leucaena leucocephala* (Lam) de Wit seeds. Heliyon.

[39] Vo T.P., Pham N.D., Pham T.V., Nguyen H.Y., Vo L.T.V., Tran T.N.H., Tran T.N., Nguyen D.Q. (2023). Green extraction of total phenolic and flavonoid contents from mangosteen (*Garcinia mangostana* L.) rind using natural deep eutectic solvents. Heliyon.

[40] Ghiasvand M.K., Heydarinasab A., Shahriari S., Fouladitajar A. (2025). Ultrasound-assisted extraction of trans-anethole from fennel seeds using deep eutectic solvents: Insights into optimization, yield, and kinetic modeling. J. Mol. Liq..

[41] Nabi P., Heydarinasab A., Shahriari S., Vaziri Yazdi A. (2025). Ultrasound-assisted extraction of crocin from saffron using natural deep eutectic solvents: Cryogenic grinding technology, yield optimization, and kinetic analysis. Food Bioprocess Technol..

[42] Soukaina K., Safa Z., Soukaina H., Hicham C., Bouchra C. (2024). Choline chloride-based deep eutectic solvents (NADES): Potential use as green extraction media for polyphenols from *Mentha pulegium*, antioxidant activity, and antifungal activity. Microchem. J..

[43] Airouyuwa J.O., Mostafa H., Riaz A., Maqsood S. (2022). Utilization of natural deep eutectic solvents and ultrasound-assisted extraction as green extraction technique for the recovery of bioactive compounds from date palm (*Phoenix dactylifera* L.) seeds: An investigation into optimization of process parameters. Ultrason. Sonochem..

[44] Wang H.X., Yang H., Nie S.M., Han X., Chang Y.H., Xu J., Nie C.D., Fu Y.J. (2024). Efficient extraction of flavonoid compounds in sweet tea (*Lithocarpus litseifolius* (Hance) Chun) via ultrasonic-natural deep eutectic solvent composite approach based on machine learning. Ind. Crops Prod..

[45] Gan Y.X., Wang C.Y., Xu C.F., Zhang P.P., Chen S.T., Tang L., Zhang J.B., Zhang H.H., Jiang S.H. (2023). Simultaneous extraction of crocin and geniposide from gardenia fruits (*Gardenia jasminoides* Ellis) by probe-type ultrasound-assisted natural deep eutectic solvents and their inhibition effects on low density lipoprotein oxidation. Ultrason. Sonochem..

[46] Hussain S., Akram H., Fida H., Butt T.E. (2024). Enviro-economic sustainability of ivy leaf powder extraction process based on phyto-geographic variation of hederacoside C in *Hedera helix* L. leaves: A lab scale study. Sustain. Chem. Pharm..

[47] Huang H., Zhu Y., Fu X., Zou Y., Li Q., Luo Z. (2022). Integrated natural deep eutectic solvent and pulse-ultrasonication for efficient extraction of crocins from gardenia fruits (*Gardenia jasminoides* Ellis) and its bioactivities. Food Chem..

[48] Olfat A., Mostaghimet T., Shahriari S., Salehifar M. (2024). Extraction of bioactive compounds of *Hypnea flagelliformis* by ultrasound-assisted extraction coupled with natural deep eutectic solvent and enzyme inhibitory activity. Algal Res..

[49] Amoroso R., Hollmann F., Maccallini C. (2021). Choline chloride-based DES as solvents/catalysts/chemical donors in pharmaceutical synthesis. Molecules.

[50] Wang W., An M., Zhao G., Wang Y., Yang D., Zhang D., Zhao L., Han J., Wu G., Bo Y. (2023). Ultrasonic-assisted customized natural deep eutectic solvents extraction of polyphenols from *Chaenomeles speciosa*. Microchem. J..

[51] Zannou O., Koca I. (2022). Greener extraction of anthocyanins and antioxidant activity from blackberry (*Rubus* spp.) using natural deep eutectic solvents. LWT-Food Sci. Technol..

[52] Tobar-Delgado E., Mejía-España D., Osorio-Mora O., Serna-Cock L. (2023). Rutin: Family farming products’ extraction sources, industrial applications and current trends in biological activity protection. Molecules.

[53] Liu Y., Li R., Wang Q., Liu X., Gong Z. (2025). Polyphenols and flavonoids in water caltrop shells from different producing areas of China: Identification, green extraction and quantitative analysis. Ind. Crops Prod..

[54] Yu X., Zhao Z., Yan X., Xie J., Yu Q., Chen Y. (2023). Extraction optimization of tea saponins from *Camellia oleifera* seed meal with deep eutectic solvents: Composition identification and properties evaluation. Food Chem..

[55] Sa E.J., Lee B.S., Park B.H. (2023). Separation of n-hexane-ethanol azeotropic mixture using choline chloride + 1,4-butanediol deep eutectic solvents. Chem. Eng. Res. Des..

[56] Didion Y.P., Tjalsma T., Malankowska M., Su Z., Matos M., Pinelo M., Crespo J., Brazinha C. (2025). Zero-waste extraction of polyhydroxy(butyrate-co-valerate) (PHBV) from mixed cultures using natural deep eutectic solvents (NADES): Unlocking the roles of molecular interactions, polarity, and viscosity. Chem. Eng. J..

[57] Koh Q.Q., Chew Z.L., Zhao Y., Kua Y.L., Gan S., Tan K.W., Lee T.Z., Lau H.L. (2024). Formulation and characterization of natural deep eutectic solvents (NADES) for simultaneous phenolics and carotenes extraction from fresh oil palm leaf. Food Bioprod. Process..

[58] Wang Z.W., Wang D.D., Fang J.X., Song Z.X., Geng J.M., Zhao J.F., Fang Y.F., Wang C.T., Li M. (2024). Green and efficient extraction of flavonoids from *Perilla frutescens* (L.) Britt. leaves based on natural deep eutectic solvents: Process optimization, component identification, and biological activity. Food Chem..

[59] Carreón-Hidalgo J.P., Ruiz-Peralta M.L., Martínez-Ramos T., Corona-Jiménez E. (2025). Natural deep eutectic solvents (NADES) based on fructose, sorbitol and xylitol for phenolic compounds extraction from moringa leaves (*Moringa oleifera*): Optimization, characterization, and modeling. Food Humanit..

[60] Abdelrahman R., Hamdi M., Baba W.N., Hassan H.M., Maqsood S. (2023). Synergistic combination of natural deep eutectic solvents and green extraction techniques for the valorization of date palm leaves: Optimization of the solvent-biomass interaction. Microchem. J..

[61] Maimulyanti A., Nurhidayati I., Mellisani B., Putri F.A.R., Puspita F., Prihadi A.R. (2023). Development of natural deep eutectic solvent (NADES) based on choline chloride as a green solvent to extract phenolic compound from coffee husk waste. Arab. J. Chem..

[62] Barbieri J.B., Goltz C., Cavalheiro F.B., Toci A.T., Igarashi-Mafra L., Mafra M.R. (2020). Deep eutectic solvents applied in the extraction and stabilization of rosemary (*Rosmarinus officinalis* L.) phenolic compounds. Ind. Crops Prod..

[63] Phaisan S., Makkliang F., Putalun W., Sakamoto S., Yusakul G. (2021). Development of a colorless *Centella asiatica* (L.) Urb. extract using a natural deep eutectic solvent (NADES) and microwave-assisted extraction (MAE) optimized by response surface methodology. RSC Adv..

[64] Tian Y., Sun D.W., Zhu Z. (2022). Development of natural deep eutectic solvents (NADESs) as anti-freezing agents for the frozen food industry: Water-tailoring effects, anti-freezing mechanisms and applications. Food Chem..

[65] Wu K., Zhang H., Lou X., Wu X., Wang Y., Zhao K., Du X., Xia X. (2024). Analysis of NADES and its water tailoring effects constructed from inulin and L-proline based on structure, physicochemical and antifreeze properties. Int. J. Biol. Macromol..

[66] Sakurai Y.C.N., Pires I.V., Ferreira N.R., Moreira S.G.C., Silva L.H.M.D., Rodrigues A.M.D.C. (2024). Preparation and characterization of natural deep eutectic solvents (NADESs): Application in the extraction of phenolic compounds from araza pulp (*Eugenia stipitata*). Foods.

[67] Gabriele F., Chiarini M., Germani R., Tiecco M., Spreti N. (2019). Effect of water addition on choline chloride/glycol deep eutectic solvents: Characterization of their structural and physicochemical properties. J. Mol. Liq..

[68] Filip D., Macocinschi D., Balan-Porcarasu M., Varganici C.D., Dumitriu R.P., Peptanariu D., Tuchilus C.G., Zaltariov M.F. (2022). Biocompatible self-assembled hydrogen-bonded gels based on natural deep eutectic solvents and hydroxypropyl cellulose with strong antimicrobial activity. Gels.

[69] Rashid R., Wani S., Manzoor S., Masoodi F.A., Dar M. (2023). Green extraction of bioactive compounds from apple pomace by ultrasound-assisted natural deep eutectic solvent extraction: Optimisation, comparison and bioactivity. Food Chem..

[70] Niu L., Zhang S., Si X., Fang Y., Wang S., Li L., Sheng Z. (2025). Ultrasonic-assisted extraction of luteolin from peanut shells using ionic liquid and its molecular mechanism. Ultrason. Sonochem..

[71] Fu X., Wang D., Belwal T., Xu Y., Li L., Luo Z. (2021). Sonication-synergistic natural deep eutectic solvent as a green and efficient approach for extraction of phenolic compounds from peels of *Carya cathayensis* Sarg. Food Chem..

[72] Hong S.M., Kamaruddin A.H., Nadzir M.M. (2023). Sustainable ultrasound-assisted extraction of polyphenols from *Cinnamomum cassia* bark using hydrophilic natural deep eutectic solvents. Process Biochem..

[73] Hou M., Hu W., Wang A., Xiu Z., Shi Y., Hao K., Sun X., Cao D., Lu R., Sun J. (2019). Ultrasound-assisted extraction of total flavonoids from *Pteris cretica* L.: Process optimization, HPLC analysis, and evaluation of antioxidant activity. Antioxidants.

[74] Hu R.S., Yu L., Zhou S.Y., Zhou H.F., Wan H.T., Yang J.H. (2023). Comparative study on optimization of NADES extraction process by dual models and antioxidant activity of optimum extraction from chuanxiong-Honghua. LWT-Food Sci. Technol..

[75] Dey S., Rathod V.K. (2013). Ultrasound assisted extraction of β-carotene from *Spirulina platensis*. Ultrason. Sonochem..

[76] Vo T.P., Nguyen T.H.P., Nguyen V.K., Dang T.C.T., Nguyen L.G.K., Chung T.Q., Vo T.T.H., Nguyen D.Q. (2024). Extracting bioactive compounds and proteins from *Bacopa monnieri* using natural deep eutectic solvents. PLoS ONE.

[77] Sulejmanović M., Panić M., Redovniković I.R., Milić N., Drljača J., Damjanović A., Vidović S. (2025). Sustainable isolation of ginger (*Zingiber officinale*) herbal dust bioactive compounds with favorable toxicological profile employing natural deep eutectic solvents (NADES). Food Chem..

[78] Hou Y.J., Wang P.W., Zhang H., Fan Y.Y., Cao X., Luo Y.Q., Li Q., Njolibimi M., Li W.J., Hong B. (2024). A high-permeability method for extracting purple yam saponins based on ultrasonic-assisted natural deep eutectic solvent. Food Chem..

[79] Shehzad K., Waheed B., Shehzad A., Ahmad M., Meng S., Jing J., Chen M., Xie M., Xu Y. (2025). Modified waste orange peels biomass residues for sustainable and promising As(V) removal: Insights into batch and column adsorption experiments and Box-Behnken design (BBD) analysis. Colloids Surf. A Physicochem. Eng. Asp..

[80] Qi H., Fu W., Liu Y., Bai J., Wang R., Zou G., Shen H., Cai Y., Luo A. (2025). Electron beam irradiation coupled ultrasound-assisted natural deep eutectic solvents extraction: A green and efficient extraction strategy for proanthocyanidin from walnut green husk. Food Chem..

[81] Beeler N., Hühn T., Rohn S., Colombi R. (2024). Development and optimization of a cocoa extraction treatment by means of the response surface methodology (RSM) and artificial neural networks (ANN). Ind. Crops Prod..

[82] Fan X.H., Zhang X.Y., Zhang Q.A., Zhao W.Q., Shi F.F. (2019). Optimization of ultrasound parameters and its effect on the properties of the activity of beta-glucosidase in apricot kernels. Ultrason. Sonochem..

[83] Sukor N.F., Jusoh R., Kamarudin N.S., Halim N.A., Sulaiman A.Z., Abdullah S.B. (2020). Synergistic effect of probe sonication and ionic liquid for extraction of phenolic acids from oak galls. Ultrason. Sonochem..

[84] Ismail B.B., Guo M.M., Pu Y.F., Wang W.J., Ye X.Q., Liu D.H. (2019). Valorisation of baobab (*Adansonia digitata*) seeds by ultrasound assisted extraction of polyphenolics: Optimisation and comparison with conventional methods. Ultrason. Sonochem..

[85] Sayem A.S., Ahmed T., Mithun M.U., Rashid M., Rana M.R. (2024). Optimising ultrasound-assisted extraction conditions for maximising phenolic, flavonoid content and antioxidant activity in hog plum peel and seed: A response surface methodology approach. J. Agric. Food Res..

[86] Oprescu E.E., Enascuta C.E., Radu E., Ciltea-Udrescu M., Lavric V. (2022). Does the ultrasonic field improve the extraction productivity compared to classical methods—Maceration and reflux distillation?. Chem. Eng. Process. Process Intensif..

[87] Pogorzelska-Nowicka E., Hanula M., Pogorzelski G. (2024). Extraction of polyphenols and essential oils from herbs with green extraction methods-An insightful review. Food Chem..

[88] Ma W., Tang M., Li S., Ma Y., Ling M., Sheng W. (2025). The effect of hydrogen bonding strength in natural deep eutectic solvents on the extraction efficiency of polyphenols. Microchem. J..

[89] Al Ragib A., Alanazi Y.M., El-Harbawi M., Yin C.Y., Khiari R. (2024). Sustainable reuse of date palm biomass via extraction of cellulose using natural deep eutectic solvent (NaDES) and microwave-assisted process. Int. J. Biol. Macromol..

[90] Chemat F., Rombaut N., Sicaire A.G., Meullemiestre A., Fabiano-Tixier A.S., Abert-Vian M. (2017). Ultrasound assisted extraction of food and natural products: Mechanisms, techniques, combinations, protocols and applications. A review. Ultrason. Sonochem..

[91] Peng Z., Wang Y., Li W., Zhan B., Zhu L., Yang D., Li G., Zhang L., Zhao Z. (2025). Ultrasonic-assisted extraction of flavonoids from *Amomum villosum* Lour. using natural deep eutectic solvent: Process optimization, comparison, identification, and bioactivity. Ultrason. Sonochem..

[92] Seo C.S., Chun J., Song K.H. (2025). Simultaneous component analysis of *Akebia quinata* seeds (Lardizabalaceae) by ultra-performance liquid chromatography-tandem mass spectrometry for quality and cytotoxicity assessment. Plants.

[93] Belmehdi O., Taha D., Abrini J., Ming L.C., Khalid A., Abdalla A.N., Algarni A.S., Hermansyah A., Bouyahya A. (2023). Anticancer properties and mechanism insights of α-hederin. Biomed. Pharmacother..

[94] Villarini M., Acito M., di Vito R., Vannini S., Dominici L., Fatigoni C., Pagiotti R., Moretti M. (2021). Pro-apoptotic activity of artichoke leaf extracts in human HT-29 and RKO colon cancer cells. Int. J. Environ. Res. Public Health.

[95] Guon T.E., Chung H.S. (2016). Hyperoside and rutin of Nelumbo nucifera induce mitochondrial apoptosis through a caspase-dependent mechanism in HT-29 human colon cancer cells. Oncol. Lett..

[96] Zanke B.W., Lee C., Arab S., Tannock I.F. (1998). Death of tumor cells after intracellular acidification is dependent on stress-activated protein kinases (SAPK/JNK) pathway activation and cannot be inhibited by Bcl-2 expression or interleukin 1β-converting enzyme inhibition. Cancer Res..

[97] Vijayakumar A., Chen B., Alam M.J., Park J.H., Park C., Nah S.Y., Kim J.H. (2025). Gintonin from *Panax ginseng* induces apoptosis in HT-29 colon cancer cells via the AMPK signaling pathway. J. Herb. Med..

[98] Yalcinkaya T., Carhan A. (2025). Investigation of natural diphtheria toxin as a potential anticancer agent in colorectal cancer: An in vitro analysis on HT-29 cells. Toxicon.

[99] Wu T.Y., Chen C.C., Lin J.Y. (2025). Caffeic acid and 5-caffeoylquinic acid inhibit HT-29 colorectal cancer cell growth through immunotherapy by inducing the Bax/Bcl-2 apoptotic pathway in vitro. Bioorg. Chem..

[100] Kim H., Xue X. (2020). Detection of total reactive oxygen species in adherent cells by 2′,7′-dichlorodihydrofluorescein diacetate staining. J. Vis. Exp..

