# Supplementary material for: Sustainable Extraction of Hedera helix Bioactive Compounds via Synergy of Natural Deep Eutectic Solvent and Ultrasound: Process Optimization, Mechanistic Insights and Anti-Colon Cancer Activity
Source: Int J Mol Sci. 2026 Feb 28;27(5):2295. doi: 10.3390/ijms27052295 (PMC12984837; doi:10.3390/ijms27052295)

Supplementary Table S1. Comprehensive formulation and preparation parameters for the synthesized NADES systems.

No	Acronyms	HBA	HBD-1	HBD-2	Molar ratio (HBA:HBD-1:HBD-2)
1	ChCl-Ur (1:2)	ChCl	Ur	-	1:2
2	ChCl-Am (1:2)	ChCl	Am	-	1:2
3	ChCl-Ca (1:2)	ChCl	Ca	-	1:2
4	ChCl-Lac (1:2)	ChCl	Lac	-	1:2
5	ChCl-1,2-Pg (1:2)	ChCl	1,2-Pg	-	1:2
6	ChCl-1,4-BDO (1:2)	ChCl	1,4-BDO	-	1:2
7	ChCl-EG (1:2)	ChCl	EG	-	1:2
8	ChCl-DMU (1:2)	ChCl	DMU	-	1:2
9	Bet-Mal (1:2)	Bet	Mal	-	1:2
10	Bet-DMU (1:2)	Bet	DMU	-	1:2
11	Bet-Ur-1,4-BDO (1:2:2)	Bet	Ur	1,4-BDO	1:2:2
12	Bet-1,4-BDO-Mal (1:2:2)	Bet	1,4-BDO	Mal	1:2:2
13	Mal-Ur-1,4-BDO (1:2:2)	Mal	Ur	1,4-BDO	1:2:2
14	Mal-DMU-1,4-BDO (1:2:2)	Mal	DMU	1,4-BDO	1:2:2
15	ChCl-DMU-1,4-BDO (1:2:2)	ChCl	DMU	1,4-BDO	1:2:2

*A dash (–) in the HBD-2 column indicates that no second hydrogen bond donor was incorporated in the NADES formulation.

Supplementary Figure S1. Chemical structures of eight bioactive compounds from *Hedera helix* L.

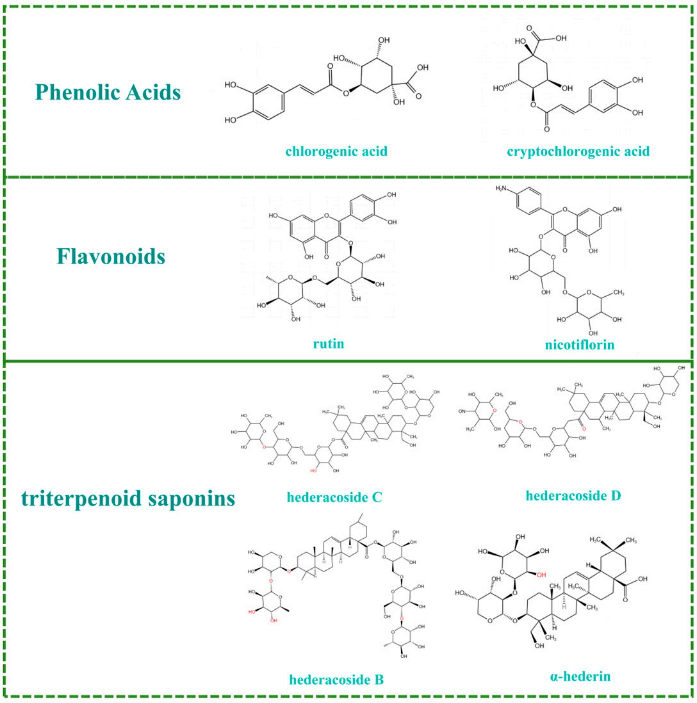

Supplementary Figure S2. Kamlet-Taft solvation parameter value of different NADESs.

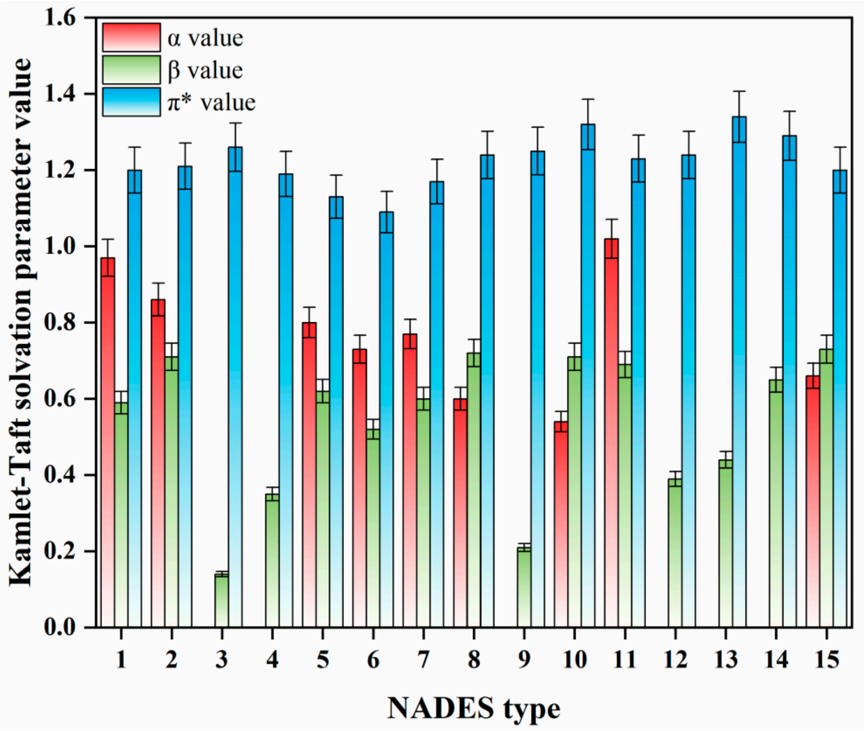

Supplementary Figure S3. Comparative analysis of FT-IR spectral features of the eutectic system NADES1-NADES13 and its molecular constituents.

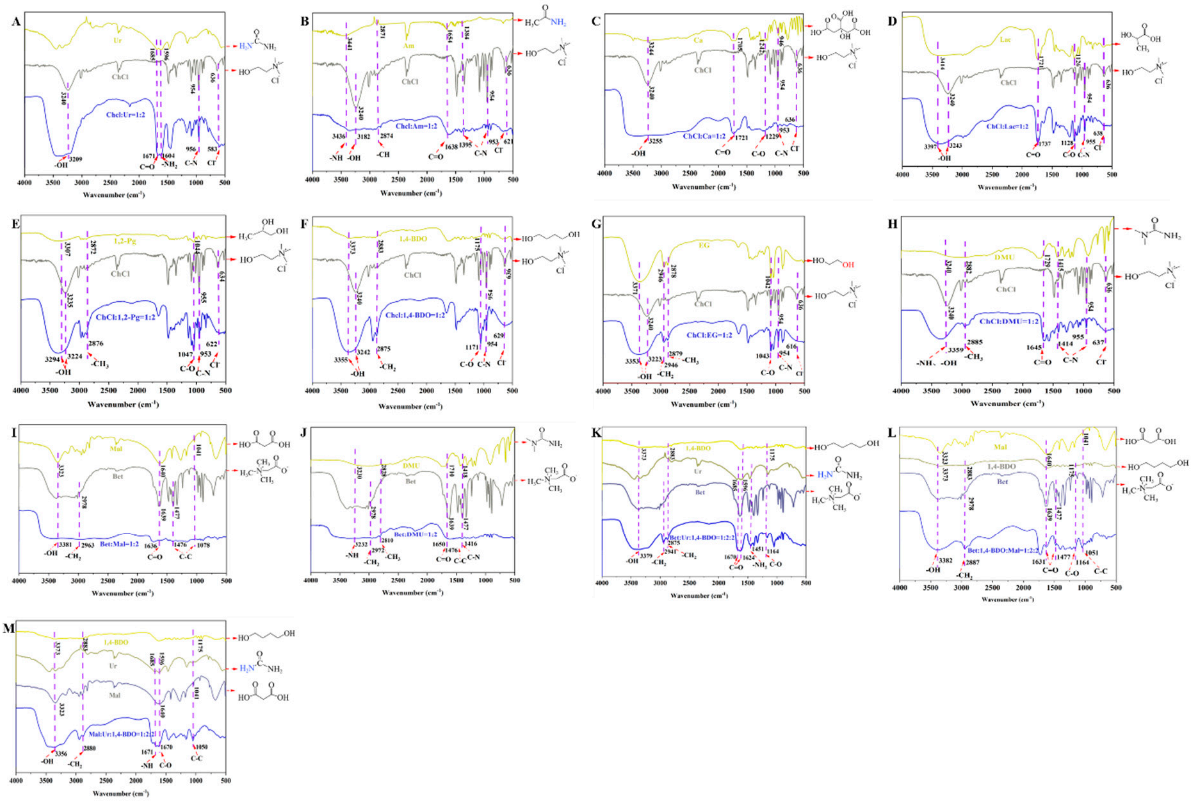

Supplementary Figure S4. Comparative analysis of FT-IR spectral features of the eutectic system NADES15 and its molecular constituents (A) and ChCl: DMU: 1,4-BDO at different molar ratios (B).

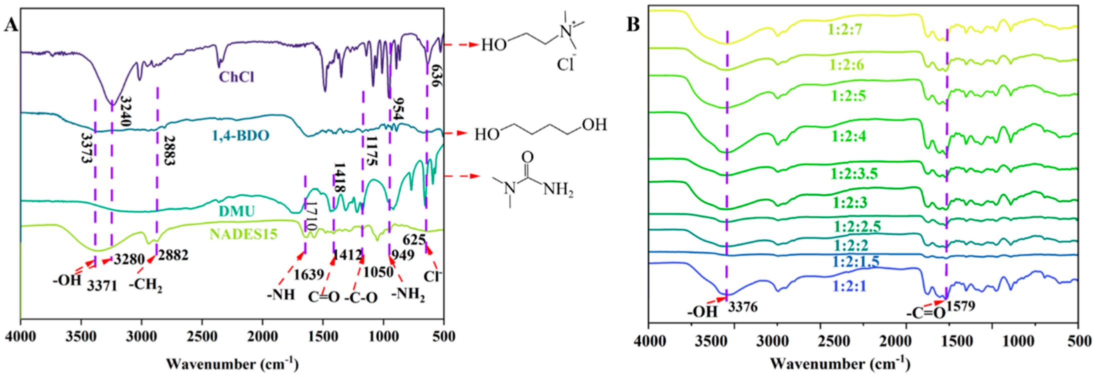

Supplement: Supplementary file 1 [file ijms-27-02295-s001.zip › ijms-4170581-supplementary.pdf]
